# Resolving the Structural Duality of Graphene Grain Boundaries

**DOI:** 10.1002/adma.202510899

**Published:** 2025-10-14

**Authors:** Haojie Guo, Emiliano Ventura‐Macías, Mariano D. Jiménez‐Sánchez, Nicoleta Nicoara, Pierre Mallet, Jean‐Yves Veuillen, Vincent T. Renard, Antonio J. Martínez‐Galera, Pablo Pou, Julio Gómez‐Herrero, Rubén Pérez, Iván Brihuega

**Affiliations:** ^1^ Departamento de Física de la Materia Condensada Universidad Autónoma de Madrid C. Francisco Tomás y Valiente, 7 Madrid 28049 Spain; ^2^ Departamento de Física Teórica de la Materia Condensada Universidad Autónoma de Madrid C. Francisco Tomás y Valiente, 7 Madrid 28049 Spain; ^3^ International Iberian Nanotechnology Laboratory (INL) Av. Mestre José Veiga s/n Braga 4715‐330 Portugal; ^4^ Université Grenoble Alpes Institut NEEL Grenoble 38000 France; ^5^ Centre National de la Recherche Scientifique (CNRS) Institut NEEL Grenoble 38000 France; ^6^ Univ. Grenoble Alpes CEA Grenoble INP PHELIQS IRIG Grenoble 38000 France; ^7^ Departamento de Física de Materiales Universidad Autónoma de Madrid C. Francisco Tomás y Valiente, 7 Madrid 28049 Spain; ^8^ Instituto Nicolás Cabrera (INC) Universidad Autónoma de Madrid C. Francisco Tomás y Valiente, 7 Madrid 28049 Spain; ^9^ Condensed Matter Physics Center (IFIMAC) C. Francisco Tomás y Valiente, 7 Madrid 28049 Spain; ^10^ Present address: Donostia International Physics Center (DIPC) Manuel Lardizabal Ibilbidea, 4 San Sebastián 20018 Spain

**Keywords:** 2D materials, atomic scale imaging and manipulation, cantilever‐based ncAFM, graphene grain boundaries

## Abstract

Grain boundaries (GBs) are ubiquitous in large‐scale graphene samples, playing a crucial role in their overall performance. Due to their complexity, they are usually investigated as model structures, under the assumption of a fully relaxed interface. Here, cantilever‐based non‐contact atomic force microscopy (ncAFM) is presented as a suitable technique to resolve, atom by atom, the complete structure of these linear defects. These experimental findings reveal a richer scenario than expected, with the coexistence of energetically stable and metastable graphene GBs. Although both GBs are structurally composed of pentagonal and heptagonal rings, they can be differentiated by the irregular geometric shapes present in the metastable boundaries. Theoretical modeling and simulated ncAFM images, accounting for the experimental data, show that metastable GBs form under compressive uniaxial strain and exhibit vertical corrugation, whereas stable GBs remain in a fully relaxed, flat configuration. By locally introducing energy with the AFM tip, the possibility of manipulating the metastable GBs, driving them toward their minimum energy configuration, is shown. Notably, the high‐resolution ncAFM images reveal a clear dichotomy: while the structural distortions of metastable grain boundaries are confined to just a few atoms, their impact on graphene's properties extends over significantly larger length scales.

## Graphical abstract

Cantilever ncAFM resolves the atomic structure of grain boundaries in graphene, revealing coexisting stable and metastable types. Both contain pentagon/heptagon defects, but metastable GBs show irregular geometries. Modeling shows metastable GBs form under compression, exhibiting vertical corrugation, while stable GBs are flat. Metastable GBs can be manipulated toward stability. Their localized distortions impact properties over extended scales.

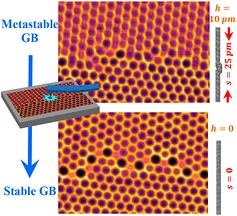

## 1 Introduction

Mechanically exfoliated graphene is probably the most perfect material ever created, with a nearly defect‐free crystalline structure. This exceptional structural quality translates into outstanding electronic, mechanical, and optical properties and an unprecedented chemical inertness.^[^ ^1^, ^2^, ^3^, ^4^, ^5^, ^6^, ^7^ ^]^ However, such crystalline perfection is not achievable at industrial scales, where large‐scale production methods inevitably introduce defects during graphene growth.^[^ ^8^, ^9^, ^10^, ^11^, ^12^, ^13^, ^14^, ^15^ ^]^ The mass production of graphene typically relies on chemical synthesis techniques, such as chemical vapor deposition (CVD), epitaxial growth on silicon carbide (SiC), and the chemical reduction of graphene oxide.^[^ ^16^, ^17^, ^18^, ^19^, ^20^, ^21^, ^22^, ^23^ ^]^ These methods yield polycrystalline graphene in which grain boundaries (GBs) disrupt the lattice structure, playing a pivotal role in the electrical, mechanical, thermal, chemical, and magnetic characteristics of the material.^[^ ^8^, ^9^, ^10^, ^17^, ^24^, ^25^, ^26^, ^27^, ^28^, ^29^, ^30^, ^31^, ^32^, ^33^, ^34^, ^35^, ^36^, ^37^, ^38^, ^39^, ^40^, ^41^ ^]^ Consequently, thorough characterization and control of these linear defects are not only essential for understanding the true properties of large‐scale produced graphene, but they also represent a remarkable opportunity to tailor and enhance its functionalities. This approach extends beyond graphene, offering similar opportunities for other non‐carbon 2D materials.

While most studies on the impact of GBs on graphene properties rely on idealized grain boundary models, consisting of alternating fully planar pentagon‐heptagon (5‐7) ring pairs in large misaligned grain angles,^[^ ^10^, ^25^, ^42^, ^43^, ^44^ ^]^ considerable efforts have been made in predicting realistic GB structures. These efforts underscore the complexity of the task, influenced by factors such as growth conditions, substrate interactions, and local stresses. In this sense, advances in experimental techniques, like transmission electron microscopy (TEM) and scanning tunneling microscopy (STM), have been crucial for accessing the atomic‐scale structure of 2D materials and characterizing their defects.^[^ ^18^, ^24^, ^26^, ^40^, ^45^, ^46^, ^47^, ^48^, ^49^, ^50^, ^51^, ^52^ ^]^ However, these methods are not without limitations. TEM typically requires freestanding and electron‐transparent samples, and the electron beam can induce structural transitions or damage, particularly in systems with delicate energy balances.^[^ ^26^, ^52^, ^53^, ^54^ ^]^ In STM, the tunneling current reflects a convolution of topographic features and the local density of states. For atomic‐scale defects in graphene, this poses a challenge, as (√3 × √3)‐R30° (R3) patterns arising from intervalley scattering processes obscure the resolution of the actual atomic structure.^[^ ^18^, ^22^, ^40^, ^55^ ^]^

Since the pioneering work by Gross et al.,^[^ ^56^ ^]^ the use of ultra‐high vacuum (UHV) non‐contact atomic force microscopy (ncAFM) with small oscillation amplitudes and tips functionalized with CO or Xe has enabled the unambiguous resolution of the chemical structure of various molecules and graphene nanoribbons.^[^ ^56^, ^57^, ^58^, ^59^, ^60^, ^61^ ^]^ However, these studies typically required systems adsorbed on metallic substrates or ultrathin insulating layers adjacent to bare metallic regions, which serve as reservoirs for individual CO or Xe molecules needed to functionalize the AFM tip.^[^ ^56^, ^57^, ^58^, ^59^, ^60^, ^61^, ^62^ ^]^ On chemically inert substrates, such as graphene, direct in‐situ functionalization is not feasible as the low reactivity of 2D material prevents the adsorption of such functionalizing entities. The alternative requires a procedure consisting of first performing ex‐situ tip functionalization with CO or Xe on a metallic substrate and then switching back to the target graphene substrate.^[^ ^63^, ^64^, ^65^ ^]^

In this work, we extend the above methodology to significantly larger modulation amplitudes (20–30 nm) ncAFM and using a tip functionalization process that results in an O‐terminated tip‐apex directly on graphene substrates (see Experimental Section). We demonstrate that cantilever‐based ncAFM can resolve atom by atom the real structure of defects in graphene layers grown on SiC(000‐1). Focusing on naturally existing GBs, our technique reveals with unprecedented resolution the intrinsic atomic structure of two types of graphene GBs on the surfaces: metastable and stable (ideal) ones. By comparing our experimental images with theoretical modeling and simulated ncAFM images, we ascribe the formation of metastable GBs to the presence of compressive strain that induces vertical corrugation. In contrast, stable GBs maintain a completely planar and relaxed structural configuration. We experimentally find that the impact of metastable grain boundaries is not confined to the boundary region itself but also alters the graphene structure over many nanometers. Additionally, we demonstrate that localized mechanical energy injection via the cantilever tip can effectively manipulate metastable GBs, driving them into a stable, flat, planar configuration.

## 2 Results

**Figure 1:**
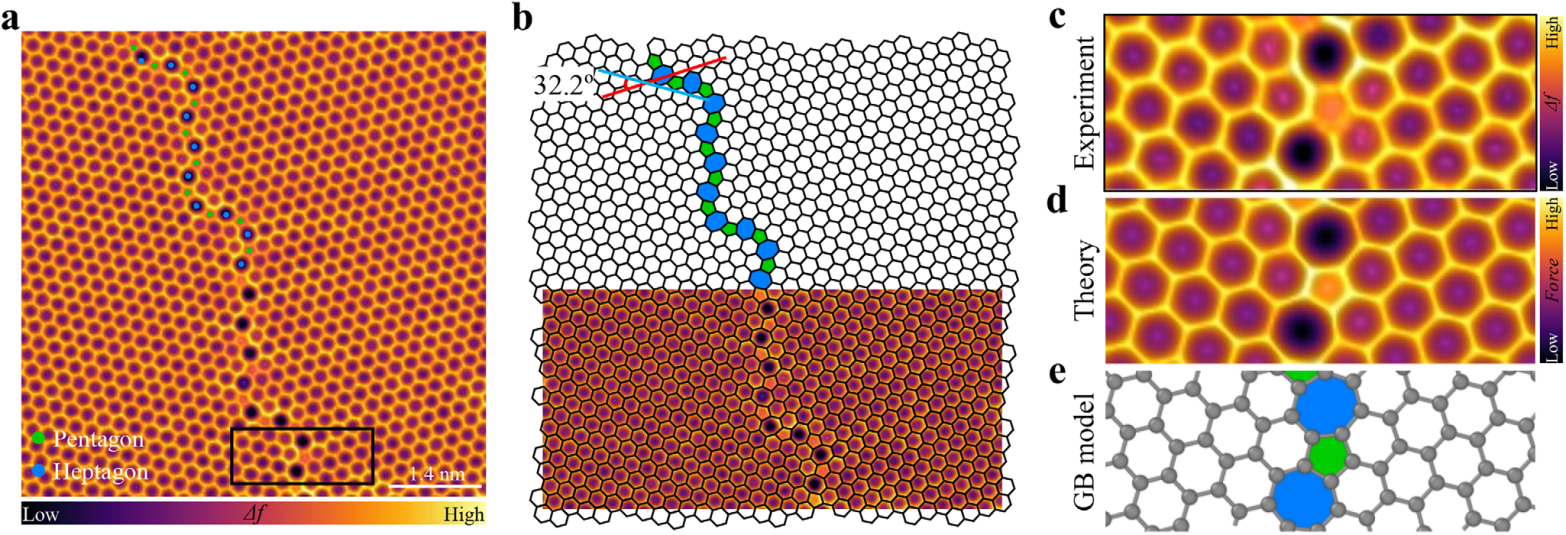
High‐resolution imaging of the atomic structure of the graphene grain boundary. a) Constant height ncAFM image showing a grain boundary region delimitating two graphene rotational domains, where the atomic structure of the GB can be inferred. FFT filtering process and 3D effect have been applied to the image. b) Schematic representation that reproduces the main characteristics of the GB shown in (a) by joining together two graphene domains with a misorientation angle of 32.2°. The experimentally measured ncAFM image depicted in (a) is superimposed below the graphene lattice for comparison. c) Magnified view within the area indicated by the black rectangle in (a). The pentagonal and heptagonal rings are clearly observed. FFT filtering process and 3D effect have been applied to the image. d) Simulated constant height ncAFM image of a GB using a CO tip and taking into consideration the effect of tip relaxation (*k* = 1.5 eV rad^−2^). A 3D effect has been applied to the image. e) Optimized atomic structure of an ideal grain boundary, which separates two graphene domains with a misorientation angle of 32.2°, used to simulate the ncAFM image shown in (d).

We first explored the use of cantilever‐based ncAFM for high‐resolution imaging of GBs on graphene surfaces (see Experimental Section for details). **Figure** **1**a shows a constant‑height ncAFM image of the boundary between two rotational graphene domains (a full‐page view is provided in Figure S1, Supporting Information). In addition to resolving the hexagonal lattices of both graphene domains, in the middle of the image, the GB region reveals an alternating sequence of pentagonal and heptagonal carbon rings. For clarity, these non‑hexagonal rings are highlighted with green (pentagons) and blue (heptagons) dots. The raw ncAFM images were processed to suppress low‑frequency moiré pattern signals, arising from the rotational misalignment between two stacked graphene layers that results in a larger‐scale interference superlattice, and thus enhance the visibility of chemical bonds (see Figures S2 and S3, Supporting Information).

The observed alternating 5–7 ring configuration aligns with theoretical predictions, which identify this motif as the lowest‐energy structural arrangement for high‐angle GBs (e.g., θ ∼ 32° as studied here).^[^ ^25^, ^42^, ^43^, ^44^ ^]^ Figure 1b illustrates a schematic model corroborating this result. It shows how a 32.2° in‐plane twist between two graphene sheets (lattice constant 2.46 Å) naturally generates the experimentally resolved 5–7 dislocation pairs. This 5–7 geometry agrees with prior studies based on aberration‐corrected transmission electron microscopy (AC‐TEM) for free‐standing graphene samples.^[^ ^26^, ^45^, ^46^, ^47^, ^49^, ^52^ ^]^ Notably, the enhanced structural clarity and resolution achieved in this work represent a significant advancement and mark a new milestone in high‐resolution imaging of C‐based nanostructures.

To gain further insights from our experimental constant height ncAFM images, we conducted direct comparisons with density functional theory (DFT) calculations for the GB structure using VASP^[^ ^66^ ^]^ and AFM image simulations using the Full Density Based Model (FDBM)^[^ ^67^ ^]^ (see also Experimental Section). Figure 1c displays a zoomed‐in view of the area marked by the black rectangle in Figure 1a. The main features in this image are accurately reproduced by the simulated image in Figure 1d (see Figure S5a, Supporting Information for simulations at different tip‐sample distances). This simulation corresponds to a fully relaxed, flat GB, with a precise 32.2° misorientation angle (see the GB model in Figure 1e; Note S2, Supporting Information for details). The rather rigid CO probe (*k* = 1.5 eV rad^−^^2^)^[^ ^58^, ^68^, ^69^ ^]^ (see also Experimental Section) used in the AFM simulations provides an accurate description of the O‐terminated tip suggested by the experimental contrast.

**Figure 2:**
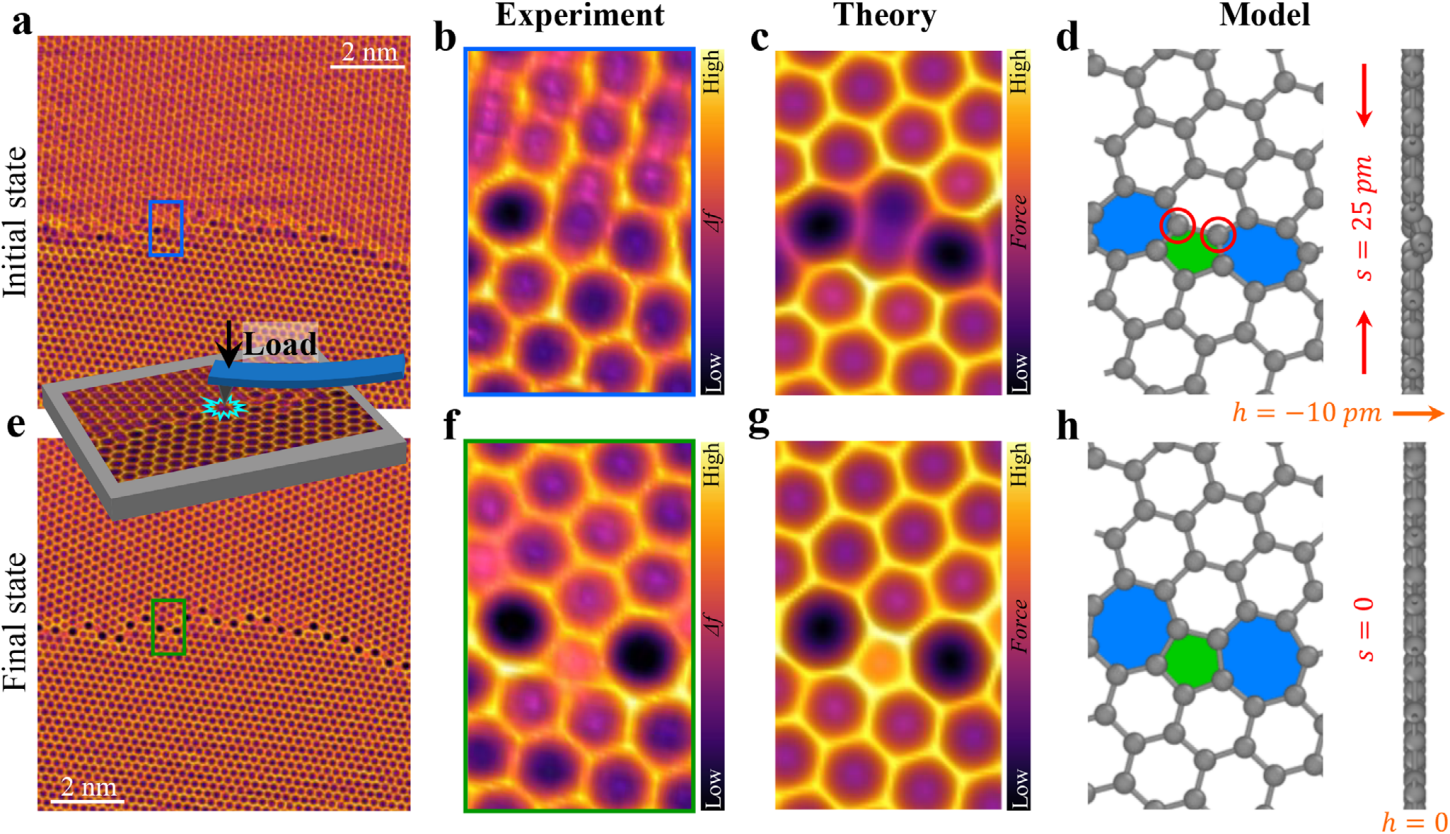
Manipulation of metastable graphene grain boundary. a,e) Corresponding constant height ncAFM images acquired in the same region before and after manipulating the GB with the ncAFM tip. Inset: 3D representation illustrating the process of grain boundary manipulation using the ncAFM tip, which consists of repeated indentation along different locations of the GB. b,f) Magnified view within the region delimited by the blue and green rectangles highlighted in (a,e), respectively. c,g) Theoretical simulated constant height ncAFM images that reproduce fairly well the feature of the grain boundary shown in the experimental images. A CO molecule with an end tip‐apex was employed in the simulation, and the effect of tip relaxation (k = 1.5 eV rad^−2^) is considered. d,h) Grain boundary model used to simulate the theoretical ncAFM images. In (d), we considered a GB under the effect of a compressive strain s = 25 pm, concentrated within the boundary region, and a vertical corrugation h = −10 pm (red cycles indicate the two lowered atoms), while in (h), we considered an ideal fully planar GB. A FFT filtering process and 3D effect have been applied to all images, except for the theoretical images, which only have a 3D effect.

The exceptional agreement between our experimental ncAFM images and DFT calculations unambiguously identifies this specific GB, intrinsic to epitaxial graphene/SiC, as an ideal, fully relaxed configuration. Moreover, this result provides a strong foundation for further exploring and characterizing other potential geometrical configurations of graphene GBs in our as‐grown samples. In **Figure** **2**a, a constant‐height ncAFM image measured in the same sample, but in a different region, shows a GB separating two graphene domains rotated by θ ∼ 31° (an enlarged view is provided in Figure S7a, Supporting Information), a similar angle as in Figure 1. A zoomed‐in view of the area indicated by the blue rectangle in Figure 2a is presented in Figure 2b. Unlike the canonical 5–7 ring arrangement in Figure 1, this boundary exhibits structural irregularity with barely visible C atoms at the interface. Atomistic simulations incorporating strain and out‐of‐plane corrugation revealed the origin of this anomaly (see Experimental Section). The optimal match, shown in Figure 2c (see Figure S5b, Supporting Information for simulations at different tip–sample distances), emerged from a compressed GB model (32.2° misorientation) with an in‐plane uniaxial strain (*s* = 25 pm), concentrated within the boundary region, inducing a strongly localized out‐of‐plane deformation (*h* = −10 pm) in the GB area. This out‐of‐plane deformation is clearly visualized in the lateral view in Figure 2d and discussed in Note S2 (Supporting Information). This specific configuration, with a 10 pm out‐of‐plane corrugation under 25 pm of in‐plane strain, was selected as it provided the best agreement with the experimental AFM images in terms of contrast and atomic‐scale features (see Figure S7, Supporting Information). The proposed model is further supported by energetic considerations from our DFT calculations (see further below).

We extended our analysis to multiple graphene regions, conclusively demonstrating that as‐grown graphene GBs on SiC coexist in both fully relaxed and strained configurations (see additional representative data in Figure S12, Supporting Information). These strained, corrugated configurations likely represent metastable GBs, where kinetic limitations during epitaxial growth hindered full structural relaxation. Crucially, this metastability suggests that targeted energy input—such as mechanical perturbation via the AFM tip—could drive these systems toward their thermodynamically favored states.

To evaluate the feasibility of this approach, we employed the AFM cantilever to locally inject mechanical energy into the metastable GBs, as shown in Figure 2a–d. We applied localized stress (indentation) by performing frequency shift vs distance (*Δf(z)*) curves at several different places along the GB area and its close vicinity (see the schematic in the inset of Figure 2; Note S3, Supporting Information). Post‐intervention ncAFM images (Figure 2e; enlarged view in Figure S7b, Supporting Information) revealed a structural reorganization within the GB, with the previously irregular heptagons transitioning to a periodic 5–7 ring pattern (see the zoomed view in Figure 2f; Note S3 (Supporting Information) for a comparison of the initial and final GB states). The final reconfigured structure quantitatively matches the simulated ncAFM image of an ideal GB (Figure 2g), which was derived from a flat, strain‑free model (Figure 2h; computational parameters are detailed in Note S2, Supporting Information). Further example of tip‐induced modification of a different metastable GB is provided in Figure S13 (Supporting Information).

**Figure 3:**
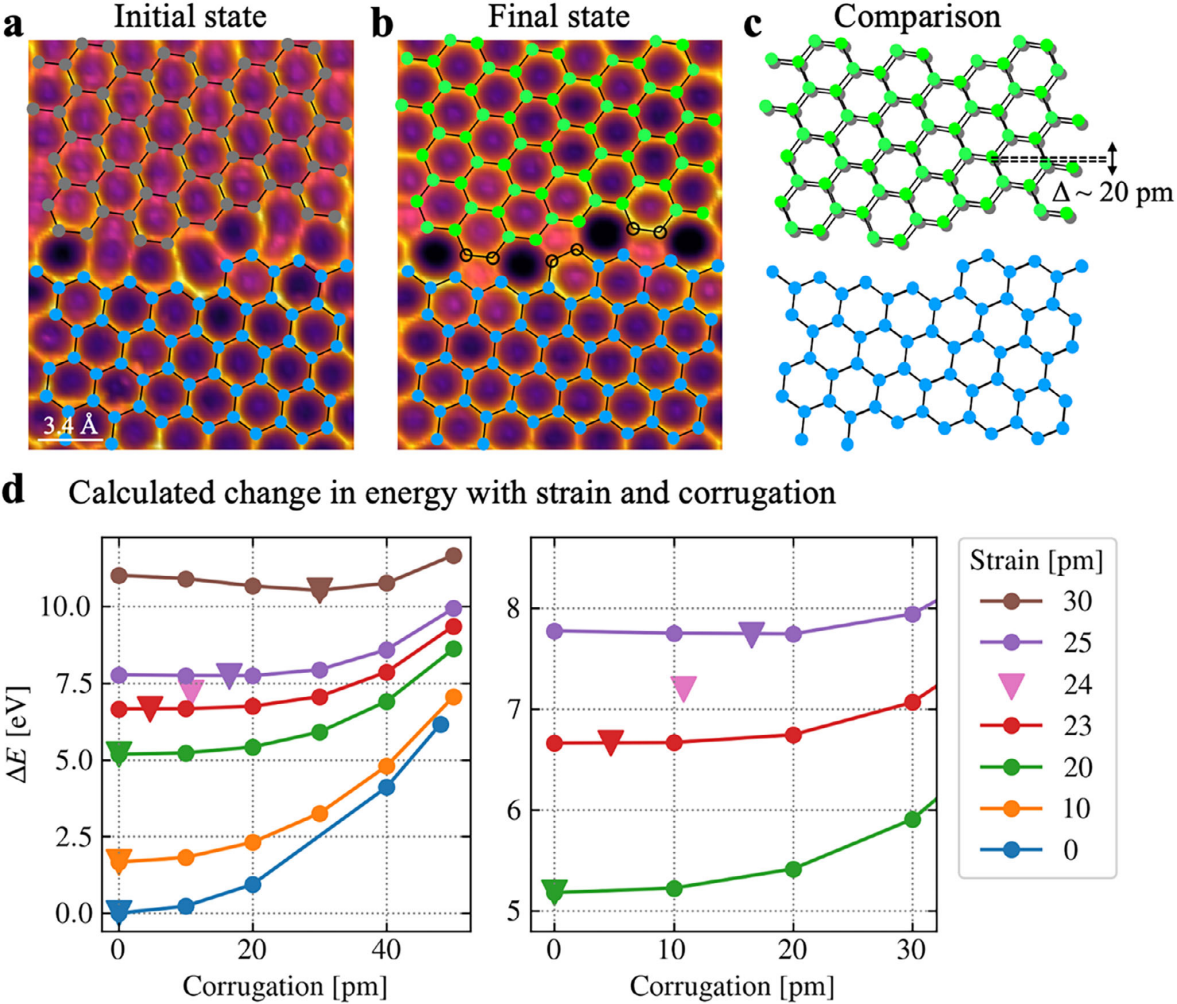
Experimental evidence of strain on the grain boundary and the expected theoretical energetic cost. a‐b) Constant height ncAFM images corresponding to the GB discussed in Figure 2a,b, respectively. Two honeycomb lattices, marking the C atom's position, are superimposed on both graphene domains in each image. Those atoms that are corrugated initially are now visible after manipulation (see the empty circles). c) Direct comparison between the graphene lattices drawn in (a‐b) before and after manipulation, from which it can be inferred that there is a rigid shift (Δ ∼ 20 pm) of the whole upper graphene lattice after manipulation. d) Calculated energy difference of the simulated GB systems (Figure S4, Supporting Information) under different corrugation (x‐axis) and uniaxial strain (color) conditions (with no‐strain and no‐corrugation as reference). The strain was introduced by rigidly displacing one grain toward the other by a distance s, while simultaneously reducing the supercell dimension along the x‐direction by the same amount. Each point is a single SCF calculation with the corresponding strain and corrugation. The inverted triangles represent the optimal corrugation (the one that minimizes the total energy) for a given strain. For a strain corresponding to s = 24 pm, only the minimized corrugation was calculated (≈10 pm).

To experimentally quantify the atomic reorganization, we analyzed the structural differences between the initial and final GB configurations by overlaying ideal graphene lattices on both sides of the GB (**Figure** **3**a–c). This comparison revealed that the as‐grown GB exhibits an in‐plane compression of ≈20 pm localized just at the frontier between both grains, impeding the formation of regular flat 5–7 motifs. The complete strain localization at the GB area is highlighted by the empty dots in Figure 3b, which correspond to the C atoms that were displaced out‐of‐plane in the original metastable GB configuration and thus were barely visible in the ncAFM image shown in Figure 3a (see also Figure S14, Supporting Information). Notably, our ncAFM measurements are sensitive to these subtle variations, allowing us to distinguish differences in the local graphene structure across the different phases. These variations may originate from small charge density asymmetries, bond order differences, further emphasizing the power of ncAFM to probe not only topography but also the underlying electronic structure.^[^ ^70^ ^]^

After the tip‐induced manipulation, the compressive strain is released in form of a rigid shift of the graphene grain above the GB, which retracted by 20 pm (see Figure 3c), while the lower grain remained nearly stationary—a phenomenon we tentatively attribute to its anchoring by macroscale surface defects, such as wrinkles and beads observed in its vicinity (see Figure S15, Supporting Information). These defects likely act as pinning sites, kinetically trapping the GB in a metastable state during epitaxial growth. Overall, these findings demonstrate that the applied mechanical energy relieves the existing stress, in this case, by rigidly retracting the upper graphene grain, enabling the metastable GB to revert to a fully flat, ideal, and energetically favored configuration. Qualitatively, this idea agrees with our DFT calculations, which indicate that the strain‐free ideal GB configuration has a lower energetic formation than the metastable one (Figure 3d). Moreover, as can be noted, our calculations show that a compressive strain of s ∼ 24 pm yields a local energy minimum for a h ∼ ‐10 pm corrugation, which further validates the proposed structural model of the metastable grain boundary discussed above.

While the exact energetic cost of converting metastable to stable GB configurations is difficult to determine (experimentally and theoretically), a qualitative estimate indicates that the nc‐AFM cantilever can supply sufficient mechanical work to induce the transformation. Under our operating conditions (k = 35 N m^−1^, A_0_ = 20nm), the cantilever stores oscillation energy in the keV range; during controlled indentation, a fraction of this energy is transferred to the surface and partially dissipated via non‐conservative processes (phonon excitation, plasticity, atomic rearrangements), see Figure S9 (Supporting Information) the characteristic *Δf(z)* data acquired during manipulation. These considerations support that localized tip‐induced input is sufficient to overcome the metastability of as‐grown GBs.

## 3 Discussion

Our theoretical simulations accurately reproduce our experimental ncAFM images of strained grain boundaries, enabling us, for example, to correlate the apparent absence of two C atoms at the edge of one grain with their downward displacement by ≈10 pm (Note S2, Supporting Information). However, it is important to note that the idealized GB model used in our simulations captures only a localized structural unit and does not reflect the more complex, irregular character of the actual grain boundary observed experimentally. In the ncAFM images, the GB displays a non‐periodic, zigzag‐like morphology, with apparent directional segments typically spanning around three graphene unit cells. This zigzag‐like morphology likely arises from local variations in strain and relaxation, which induce alternating configurations along the GB. In particular, we observe that for some segments, whose orientation is similar to the strain direction applied in our simulations, two edge atoms from one grain (e.g., the upper grain) appear to relax downward, while in adjacent segments the local strain orientation differs more substantially—deviating from the simulated strain profile—and the relaxation behavior becomes more complex or less well‐defined. This alternation leads to an effective edge relaxation over extended GB segments, with one grain exhibiting a greater number of relaxed edge atoms. Thus, this relaxation profile acts analogously to an “effective edge”, which has important implications for the electronic and atomic structure of the adjacent graphene grains. This is seen, for example, in Figures S14a and S16 (Supporting Information), where the upper grain in the strained GB displays a peculiar bond texture in which one bond per carbon hexagon has higher intensity. On the contrary, this so‐called quinoidal bond texture is not seen near the relaxed grain boundary. While the detailed description of this phenomenon is far beyond the scope of this study, we believe that an intuition of it may be found in the framework of Clar sextet theory.

Clar's theory states that the π‐electron of graphene is described by the superposition of three equivalent Clar formulas, in which every third carbon hexagon hosts a Clar sextet, preserving the overall aromatic character across the lattice.^[^ ^71^, ^72^ ^]^ The presence of defects or edges generally breaks this degeneracy, leading to a departure from the prototypical all‐benzenoid configuration.^[^ ^71^, ^72^, ^73^ ^]^ Interestingly, the grain boundary does not appear to break the aromaticity of adjacent graphene grains. Indeed, it is possible to construct Clar formulas preserving the threefold degeneracy of Clar phases on both sides of the grain boundary if the double bonds associated with its formation remain confined in it (Figure S17, Supporting Information). However, the aromaticity of the upper grain seems to be lifted in the presence of strain, as evidenced by the quinoidal bond texture. We believe this can be understood as a dramatic long‐range electronic reorganization due to a small change in the distribution of double bonds within the grain boundary, as illustrated by Clar formulas consistent with the quinoidal bond texture in Figure S18 (Supporting Information). At the moment, we do not have sufficient understanding of the details of the grain boundary to explain the selection of this particular configuration over the other possible ones. We believe that specific theoretical treatment is needed to solve this issue in the future. Similarly, we acknowledge that our current work does not provide information on the electronic properties of the observed metastable GBs, which constitute an interesting avenue to be experimentally and theoretically explored in the future.

Overall, our study provides several significant novel aspects in the characterization of the graphene surface and its GB. While previous works had determined the atomic structure of graphene GBs using TEM,^[^ ^26^, ^45^, ^46^, ^47^, ^49^, ^52^ ^]^ this work constitutes the first to demonstrate that ncAFM can also resolve these structures. This alternative approach offers several advantages: it is suitable for semiconducting and insulating substrates, does not depend on material thickness, and can be applied to bulk material samples. Second, the study demonstrated for the first time that graphene GBs can also exist under metastable configurations, which is an aspect that has been largely overlooked until now, even theoretically. In addition, it is shown that the metastable grain boundaries can be driven to an energetically stable configuration through tip‐induced manipulation. This result adds a new paradigm to the understanding of the structural properties of graphene GBs.

On the technical side, we have introduced a tip functionalization method that can be performed in situ directly on the graphene substrates to achieve high‐resolution atomic contrast imaging of its surface. While offering similar resolution output, it substantially simplifies the experimental procedure necessary in traditional ex situ techniques that require first functionalizing the tip with CO or Xe on metallic surfaces and then switching back to the target graphene sample.^[^ ^63^, ^64^, ^65^ ^]^ Additionally, given the success of O‐terminated tips in imaging polar materials like oxides,^[^ ^74^, ^75^, ^76^ ^]^ we expect that this tip conditioning could be used for high‐resolution imaging of other 2D materials like the Transition Metal Dichalcogenides (TMDs).

Finally, although earlier studies using ncAFM had resolved the atomic structure of graphene surfaces,^[^ ^64^ ^]^ none had focused specifically on grain boundaries. Moreover, these early results were obtained using tuning‐fork‐based ncAFM systems with a small modulation amplitude (<1 Å). This is in stark contrast to the cantilever‐based ncAFM system set up with a large modulation amplitude (>20 nm) employed in this work. Therefore, this work broadens the applicability and versatility of ncAFM.

## 4 Conclusion

In summary, we employed cantilever‐based ncAFM under UHV at 5 K to characterize naturally occurring grain boundaries on polycrystalline graphene. High‐resolution constant‐height imaging directly revealed two atomic‐scale GB configurations: metastable boundaries, identified by irregular pentagon–heptagon ring patterns, and ideal, fully planar relaxed boundaries. Supported by theoretical modeling and simulated ncAFM images, we showed that metastable GBs arise from local uniaxial compressive strain and vertical corrugation, while ideal GBs remain flat. By injecting localized mechanical energy with the ncAFM tip, we converted strained, metastable GBs into their stable, relaxed form. Together, these results establish ncAFM as a noninvasive, atomically precise technique for both resolving and manipulating defects in 2D materials. Importantly, metastable GBs not only introduce local scattering centers affecting charge/thermal transport or inducing local magnetic moments but also exert influence well beyond their immediate vicinity. Such long‐range effects necessitate their inclusion in realistic polycrystalline graphene models, particularly for GB‐containing devices.

## 5 Experimental Section

### Sample Preparation

The graphene samples were grown under UHV conditions by graphitization of the SiC(000‐1) surfaces (Carbon face termination) following a similar procedure described elsewhere.^[^ ^18^, ^22^ ^]^ The resulting samples were characterized by a multilayer graphene scheme, with a typical thickness of ≈3–5 layers, that featured a highly disordered rotational stacking configuration between the successive graphene layers. This led to an electronic decoupling of the π‐bands between graphene sheets. Therefore, the first surface graphene layer could be considered as ideal graphene, with the Dirac point being close to the Fermi level (neutral doping).^[^ ^77^, ^78^, ^79^ ^]^ Another characteristic of the samples used in the present work was that graphene surfaces presented different crystallographic orientations. This triggered the natural formation of grain boundaries (GBs) at the frontiers that separate two graphene rotational domains (polycrystalline surface). Such regions were identified in this work by acquiring atomically resolved ncAFM images. Additionally, as a consequence of the rotational disorder of graphene layers grown on SiC(000‐1),^[^ ^18^, ^22^ ^]^ moiré superstructures could be commonly found on the surface of Gr/SiC(000‐1) samples (Figure S19, Supporting Information).

### ncAFM Measurements

Experiments were conducted at a sample temperature of 5 K in a UHV system (base pressure ≈10^−10^ Torr) that features two interconnected chambers. One of them was used for sample cleaning and preparation, while the other hosted a custom‐built cantilever‐based combined low temperature ncAFM/STM microscope.^[^ ^80^ ^]^ An optical interferometer setup was employed to detect the dynamics of the cantilever.^[^ ^81^ ^]^ AFM measurements were performed in the frequency modulation mode, while keeping a constant oscillation amplitude. Commercially available platinum‐coated Si cantilevers (PPP‐NCLPt, Nanosensors) were used as probes for the experiments. In this work, typical values of the cantilever's dynamics are: oscillation amplitude *A* ∼ 20 nm, natural resonance frequency *f_0_* ∼ 1.7 × 10^5^ Hz^2^, quality factor *Q* = ≈6.2 × 10^5^, and cantilever stiffness *k* = 35 N m^−1^. All ncAFM images were measured either in the constant height mode or in the constant frequency shift (*Δf*) mode, as explicitly indicated on each figure. During data acquisition, a bias voltage (*V_CPD_*) was applied to the sample to minimize the long‐range electrostatic forces. STM measurements were performed under the same ncAFM experimental setup (the cantilever oscillation is kept active), but switching the feedback control channel now to the tunneling current. The topography STM images were measured in the constant current mode. All ncAFM/STM images were acquired and processed with the help of the WSxM software.^[^ ^82^ ^]^

### Tip‐Apex Preparation

Before performing high‐resolution ncAFM imaging of the chemical structure of the GBs, the tip apexes of the cantilevers were prepared on several contaminants located on the graphene surfaces (see Note S9, Supporting Information for more details). This was needed to achieve the ultimate atomic‐scale resolution shown here, which was not accessible using the bare tip apex of a commercial cantilever. This procedure differed from the standard experimental techniques reported in the literature for the acquisition of bond‐resolved images using ncAFM, which were based mainly on the controlled tip functionalization with CO molecules, and also with other inert terminations like a CuO_x_ nanocluster or an Xe atom.^[^ ^56^, ^57^, ^74^ ^]^ The contrast observed in the experiments strongly suggests an O‐terminated apex. This identification was further supported by the simulations with a rigid CO probe (see Theoretical modeling section below), which provides a striking match with the experimental results. In the present case, the presence of oxygen at the apex was tentatively attributed to some kind of metallic oxides (MO_x_) that were picked up intentionally during scanning from the contaminants present in the samples, although the ultimate chemical composition remains unknown. Nevertheless, it was noted that such uncontrolled tip‐apex functionalization could be performed on a daily basis, as it could be inferred from the multiple high‐resolution images of different grain boundaries that were measured and shown in this work. However, it was emphasized that there could be some experimental challenges and constraints when these unknown contaminants are not present in other samples in the future.

Complementary, it was also shown that high atomic resolution of the grain boundaries obtained in the constant height ncAFM imaging mode was not attainable in the topographic constant *Δf* acquisition mode (see Figure S21, Supporting Information). Similarly, it was corroborated that it was not feasible to image the chemical structure of the graphene grain boundaries studied in this work with STM (see Figure S22, Supporting Information), which was in good agreement with previous works.^[^ ^18^, ^24^, ^40^, ^48^, ^50^, ^51^ ^]^

### Theoretical Modeling

Simulations were carried out to determine the possible atomic structure of the grain boundary. These included DFT simulations to establish the energy landscape of strain and corrugation on the GB and AFM simulations to compare with observed experimental results. The structure for the 32.2° GB was adapted from the Ref. [25]. The supercell (see Figure S4, Supporting Information) included two anti‐parallel and identical grain boundaries along the y‐axis with equidistance to their periodic images.

Experimental analysis (see Figure 3c) reveals that the initial state of the boundary appears locally compressed in the direction perpendicular to the GB. This strain was confined to the boundary region, which became narrower compared to the final unstrained configuration. The complexity of the system and the difficulty in determining realistic constraints, including the effects of underlying layers, the macroscopic strain distribution, etc., prevented the use of standard DFT calculations allowing full atomic relaxation. Instead, a simplified model that captures the essential features of the system was constructed. Starting from the ideal GB geometry, uniaxial strain was introduced by rigidly displacing one grain toward the other by a distance *s*, while simultaneously reducing the supercell dimension along the x‐direction by the same amount. This approach concentrates the imposed strain within the boundary region, consistent with experimental observations. Keeping this strained configuration fixed, a set of single‐point DFT calculations was performed for different vertical corrugation values to determine the corresponding free energy landscape. Corrugation was modeled by lowering the two C atoms shared by the 5–6 pair flanked by two 7 rings. In this way, the DFT free energy for each combination of strain and corrugation was obtained.

Calculations were performed with VASP^[^ ^66^ ^]^ under the PBE‐GGA approach to the exchange‐correlation potential^[^ ^83^ ^]^ and using a PAW pseudopotential.^[^ ^84^ ^]^ The plane wave basis set was cutoff at 400 eV. Dispersion interactions were included with the DFT‐D3 correction.^[^ ^85^ ^]^ The supercell was sampled in the reciprocal space with a Monkhorst–Pack 3 × 5 × 1 k‐points grid.^[^ ^86^ ^]^ The reference energy was from the optimized grain boundary (no‐strain, no‐corrugation).

To validate the most likely structures and the experimental observations, ncAFM images were simulated under various combinations of strain and out‐of‐plane corrugation with the Full Density Based Model (FDBM).^[^ ^67^, ^87^ ^]^ This model retained the DFT accuracy in the calculation of forces with inert molecular probes and provides the computational efficiency needed to simulate the entire ncAFM image. Application to different systems showed that FDBM captures the most important features in ncAFM images, providing an accurate description of the observed contrast. These features arise from the interplay of three different force contributions: short‐range Pauli repulsion, electrostatics, and dispersion forces, together with probe deflection induced by the interaction.

Based on the observed resolution and contrast features, and the tip‐apex preparation followed in the experiments, the most probable tip configuration corresponds to a metal oxide MO_x_ apex terminated by a single oxygen atom. To approximate this configuration in simulations, a CO‐functionalized probe was employed as implemented in the well‐tested FDBM framework. This approach effectively captures the main characteristics expected from an oxygen‐terminated MO_x_ tip. In the case of metal tips functionalized with a CO molecule at the apex, the high lateral flexibility of the CO probe strongly contributed to the sharp bond contrast observed in ncAFM images at short distances.^[^ ^58^, ^60^ ^]^ To better match the experimental contrast observed with the more rigid O‐terminated MO_x_ tip, a stiffer torsional spring constant (*k*) of 1.5 eV rad^−2^ was employed, in contrast to the standard value of 0.4 eV rad^−2^ typically used for CO‐metal probes.^[^ ^67^, ^87^ ^]^ The short‐range Pauli interaction in FDBM has been calculated with parameters α = 1.06 and *V* = 35.2 eV Å^3(2α‐1)^.

## Conflict of Interest

The authors declare no competing interests.

## Supporting information



Supporting Information

## Data Availability

The data that support the findings of this study are available from the corresponding authors upon reasonable request.
