# Supplementary material for: Resolving the Structural Duality of Graphene Grain Boundaries
Source: Adv Mater. 2025 Oct 14;38(51):e10899. doi: 10.1002/adma.202510899 (PMC13568866; doi:10.1002/adma.202510899)
Supplement: Supplementary file 1 [file ADMA-38-e10899-s001.pdf]

Supporting Information for

Resolving the Structural Duality of Graphene Grain Boundaries

Haojie Guo, Emiliano Ventura-Macías, Mariano D. Jiménez-Sánchez, Nicoleta Nicoara, Pierre Mallet, Jean-Yves Veuillen, Vincent T. Renard, Antonio J. Martínez-Galera, Pablo Pou, Julio Gómez-Herrero, Rubén Pérez, Iván Brihuega**

**Corresponding authors:* haojie.guo@dipc.org; ivan.brihuega@uam.es

This PDF includes:

Supplementary note 1: Data processing of the experimental constant-height ncAFM images 2

Supplementary note 2: Theoretically modelling and simulated ncAFM images of graphene grain boundary 5

Supplementary note 3: Manipulation of the metastable graphene grain boundaries 8

Supplementary note 4: Additional data regarding the coexistence of metastable and ideal GBs, and their manipulation.. 10

Supplementary note 5: Experimental evidence of strain in the metastable grain boundary................. 11

Supplementary note 6: Morphology of the metastable grain boundary area....................................... 12

Supplementary note 7: Long range perturbation and Clar's sextet theory in graphene grain boundaries 13

Supplementary note 8: Moiré patterns on Gr/SiC(000-1) 15

Supplementary note 9: Conditioning of the experimental ncAFM tip-apex 16

Supplementary note 10: Graphene grain boundaries visualized in topographic ncAFM and STM imaging mode 17

Supplementary note 1: Data processing of the experimental constant-height ncAFM images

In this note, we disclose in detail the filtering procedure applied to some of the ncAFM images presented in the main manuscript (Figures 1–3) and in the supplementary information (e.g., Figure S1). This procedure was designed to enhance the visibility of features within the grain boundary regions, which are the central focus of this study. The approach involves a two-step method, as illustrated with the example shown in Figure S2. We begin with the experimentally acquired raw image (Figure S2a), which reveals two graphene rotational domains separated by a grain boundary region, along with superimposed moiré patterns. We first applied an FFT filtering to eliminate the low-frequency (long-wavelength in real space) signals associated with the moiré patterns (Figure S2b). Following this, a 3D illumination effect was employed to enhance contrast along the *z*-axis, thereby improving the visibility of the chemical bonds (Figure S2c). The resulting image corresponds to those shown in Figure 1 and Figure S1. By comparing the images shown in Figure S2, we observe that our data processing method minimally impacts the grain boundary (GB) region, which is the primary area of interest in this work.

As we stated above, the FFT filtering process removes only the low-frequency signals from the non-contact constant-height ncAFM images. These low-frequency components are typically linked to the moiré patterns observed on the graphene surface. Therefore, by applying FFT filtering, we effectively suppress only the moiré pattern signal contrast without altering the intrinsic features of the topmost graphene layer, as visually demonstrated in Figure S3. This approach ensures enhanced feature visibility while preserving the integrity of the GB regions.

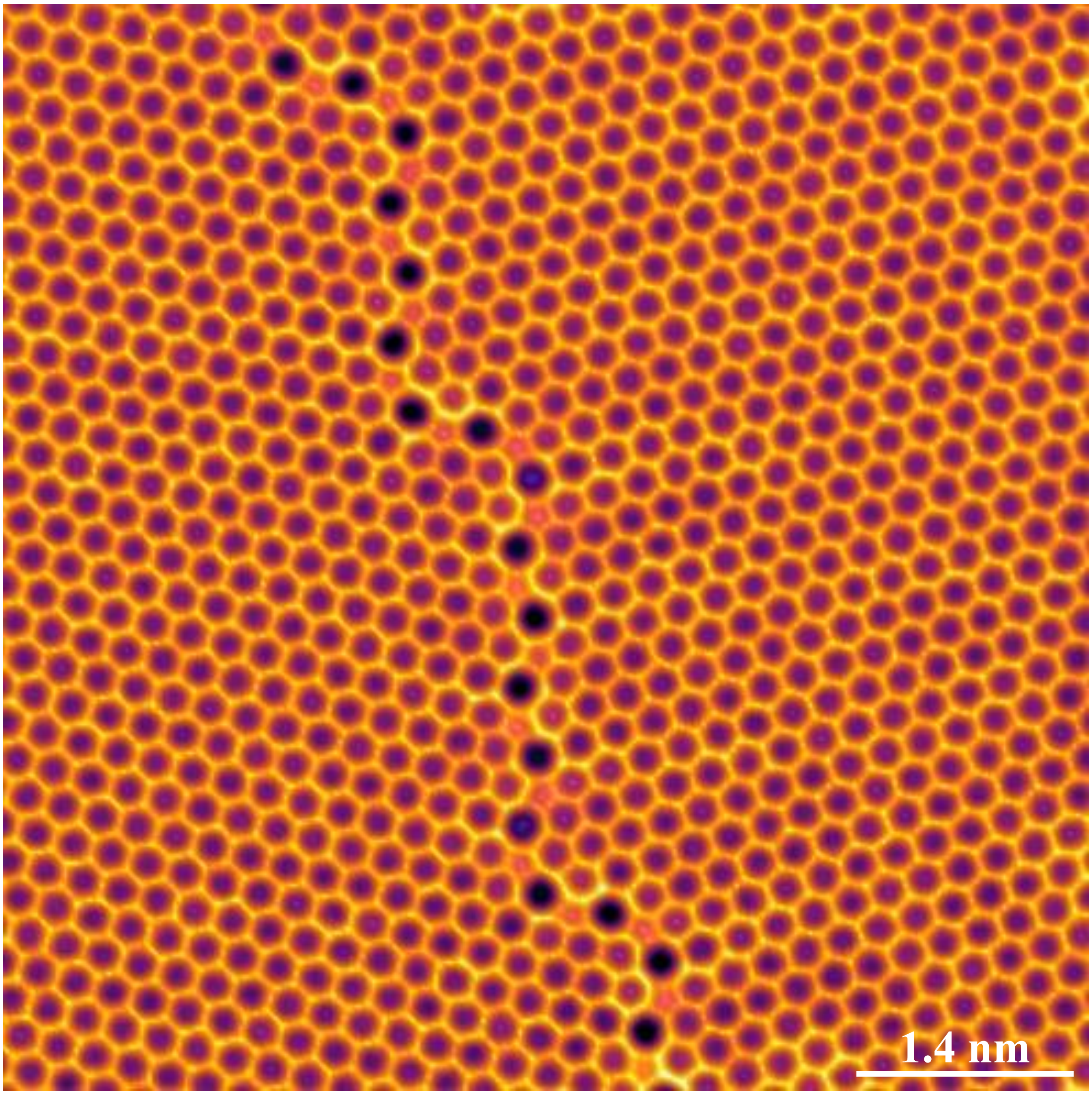

Figure S1. Enlarged view of the same constant height ncAFM image shown in Figure 1a.

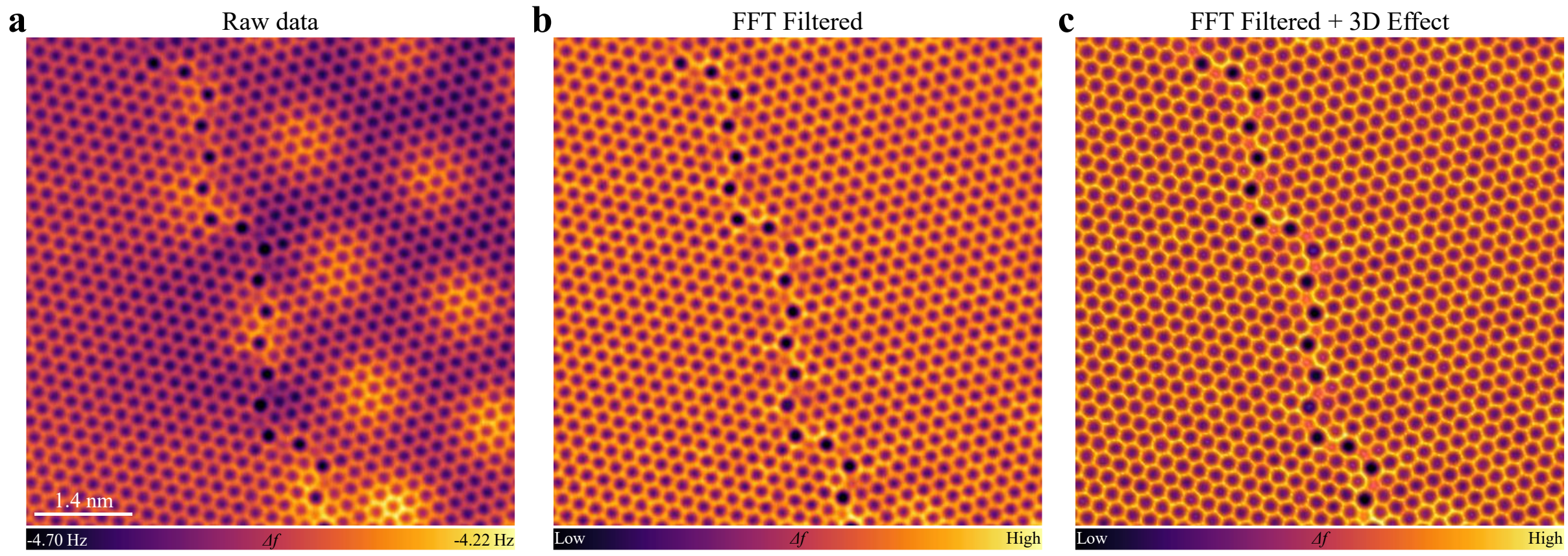

Figure S2. Example of data processing applied to the constant height ncAFM images. The experimentally measured raw data is shown in (a). By filtering out the low frequency signals of this image, we obtain the image shown in (b). By further applying a 3D effect, that is, we illuminate the image in the z-axis to enhance the visibility of the chemical bonds, we obtain the image depicted in (c).

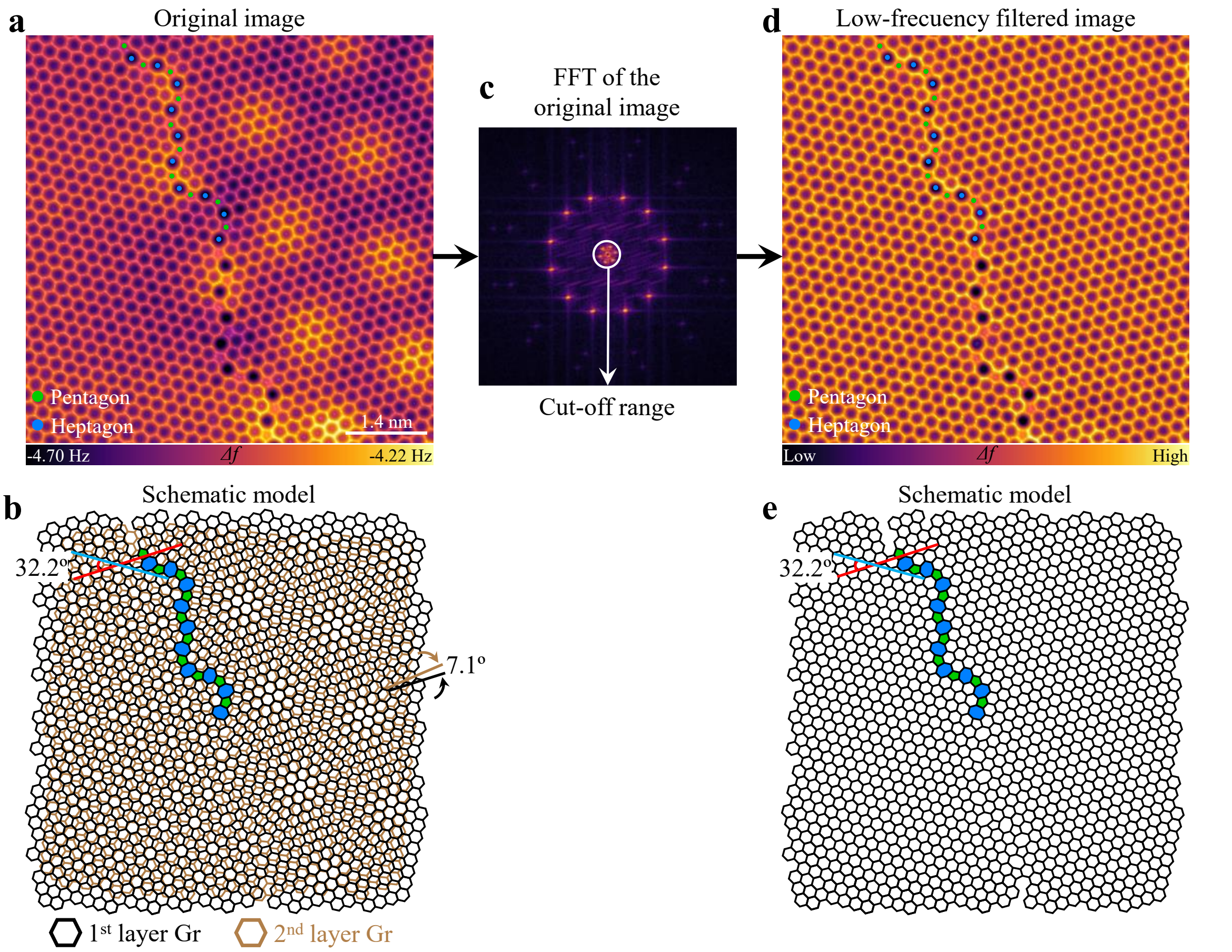

Figure S3. FFT filtering procedure applied to the ncAFM images. a) Original measured constant height ncAFM image. b) Schematic representation obtained by placing a graphene sheet, that features two rotational domains with a misorientation angle of 32.2°, on top of another graphene sheet. Both graphene layers are rotated by an angle of 7.1° with respect to the upper-right domain. This schematic model reproduces all the main characteristics depicted in (a). c) Calculated FFT pattern of the image shown in (a). Using this pattern as reference, we filtered the original image by cutting off all the signals within the white circles (low frequency signals). The result is depicted in (d), which corresponds to the image shown in Figure 1a in the main text. e) Schematic representation that reproduces the characteristics of the image (d). Therefore, our FFT filtering procedure eliminates the signal contribution coming from the bottom graphene layer, so that we can focus our attention only on the topmost graphene layer. A 3D effect has been applied to all images.

Supplementary note 2: Theoretically modelling and simulated ncAFM images of graphene grain boundary

To account for the experimental observations presented in the main text we performed theoretical modeling of graphene grain boundary. We employed two different grain boundaries models, as illustrated in Figure S4, namely the ideal relaxed GB and the strained and corrugated GB. In the case of the ideal GB (Figure S4a), we constructed it by merging two graphene sheets that present a misorientation angle of exactly 32.2º between them. The employed supercell has a size of 2.797×0.89 nm^2 and with a total number of C atoms of 96. As can be seen from the side view, the ideal GB remains in a fully flat configuration. Conversely, in the case of the strained and corrugated GB, we used the same base model as for the ideal GB, but additionally imposed a compressive uniaxial strain (s) along the x-axis, concentrated within the GB region, and explicitly enforced a vertical corrugation (h) (see Methods)

Using the above-described models, we simulated the corresponding ncAFM images using a CO molecular terminated tip-apex (see Methods section in the main text) and considering the potential impact or not of the mobility of the CO molecule on the tip-apex. For this purpose, we mimic a static CO molecular tip or consider its relaxation effect (k = 1.5 eV/rad^2), where the torsional spring constant k is intentionally set much higher than typical values used for modeling real CO-functionalized tips, in order to limit the probe lateral relaxation as expected for the oxidized apexes used in these experiments. They obtained simulated ncAFM images of the ideal and strained and corrugated GBs are shown in Figure S5 at different tip-sample distances (Z_{ts}). The theoretical images used for comparison with the experiments correspond to the simulated ncAFM images at Z_{ts} = 297 pm and considering the relaxation effect of the CO tip. The choice of this intermediate tip-distance as our reference relays in the fact that all the experimental constant height ncAFM images were recorded always in the attractive short-range force regime. Additionally, we preferred to opt for considering the CO tip relaxation effect in the simulated images as it gives qualitatively the best fit results with our experimental images, although we emphasize that, quantitatively, the difference with respect to a static tip is almost undetectable.

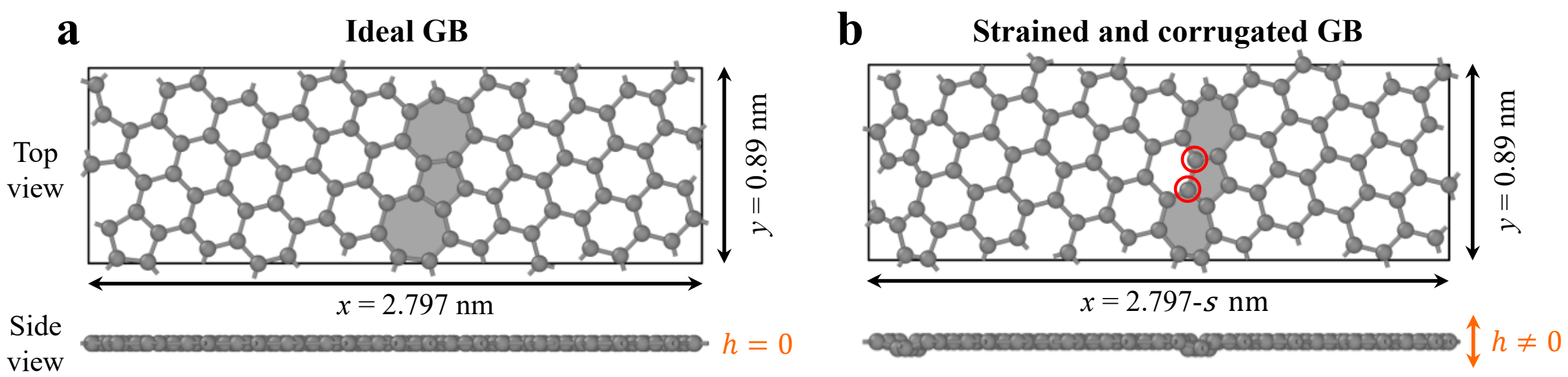

Figure S4. Grain boundary supercell used for the simulation of the ncAFM images. a) Supercell used to simulate the ideal, fully flat GB configuration. b) Supercell used to simulate a strained and corrugated GB, which is built from the ideal GB by rigidly displacing one grain toward the other by a distance s, while simultaneously reducing the supercell dimension along the x-direction by the same amount and by enforcing a vertical corrugation (h) in the two C atoms shared by the 5-6 pair flanked by two 7 rings (highlighted by the red cycles).

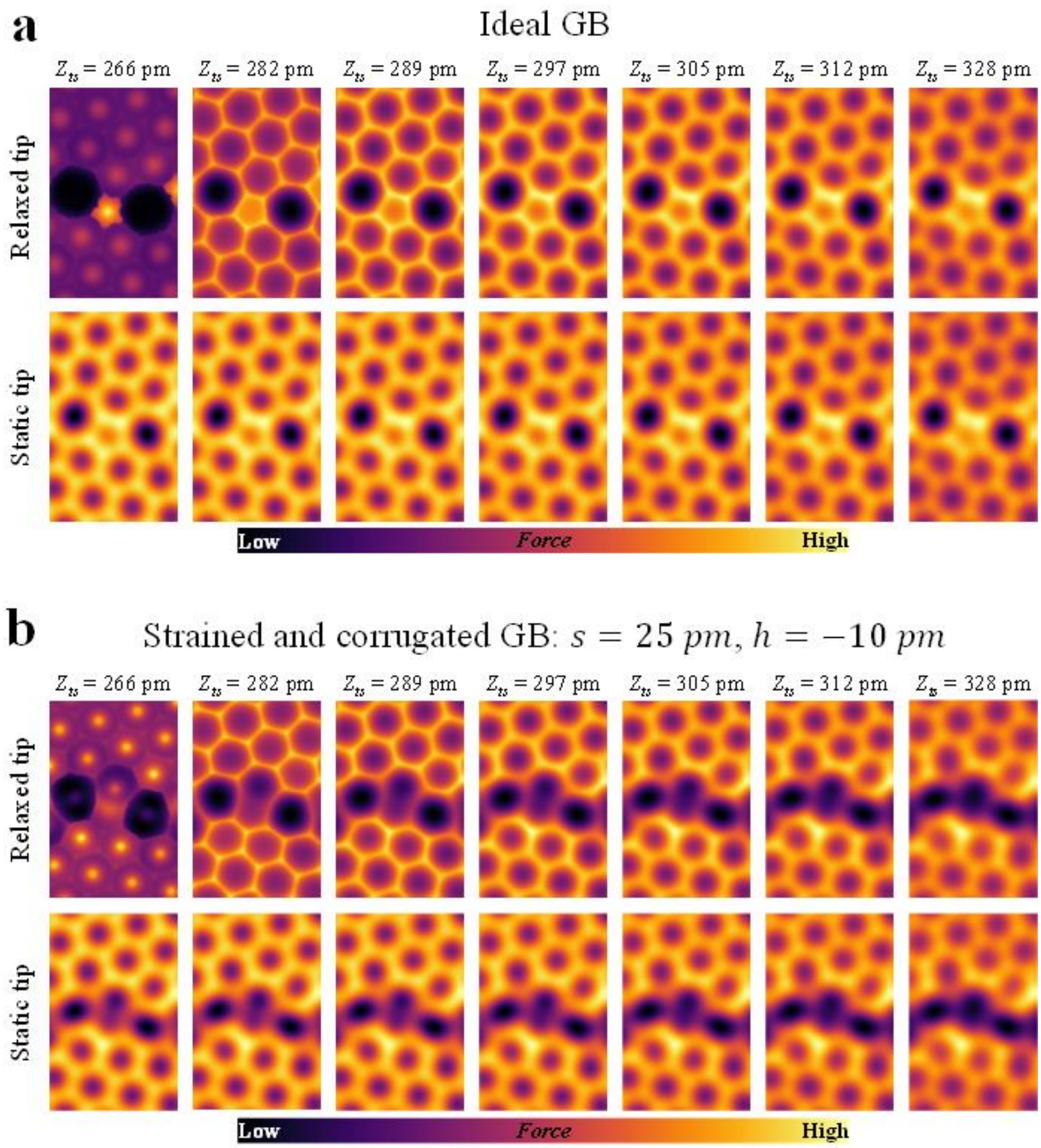

Figure S5. Simulated ncAFM images of an ideal GB (a) and a strained and corrugated GB (b) at different tip-sample distance (Z_{ts}). A CO tip was employed to simulate all the images. Also, we show, comparatively, images simulated considering the effect of tip relaxation (k = 1.5 eV/rad^2) or not (static).

In the experimental ncAFM images of strained grain boundaries, two atoms at the edge of one grain appear to be missing. This apparent absence can be explained by considering the simulated structural relaxation: the two atoms relax downward by approximately 10 pm. This vertical displacement has two concurrent effects on the ncAFM contrast (see Figure S6). First, the reduction in height directly lowers the local tip-sample Pauli repulsion, decreasing the *frequency shift* signal. Second, an increased out-of-plane localization of the corresponding C–C bond leads to a more confined electronic density, further reducing the spatial extent of the short-range interaction. As a result, these atoms contribute less contrast and may appear absent in the images.

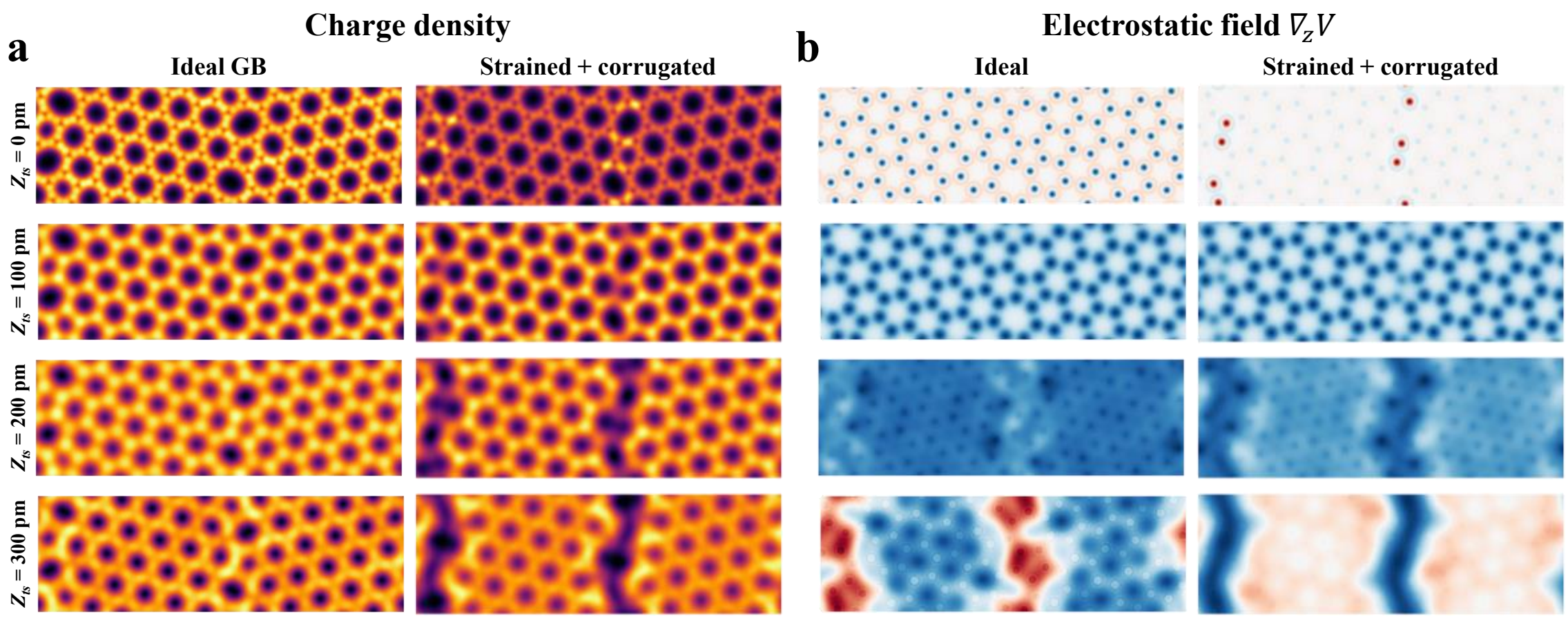

Figure S6. Simulated charge density and electrostatic field maps for ideal and strained grain boundaries. (a) Charge density maps computed at different heights $Z = 0$, 100, 200, and 300 pm for the ideal GB (left column) and the strained and corrugated GB model (right column). (b) Corresponding vertical component of the electrostatic field, $\nabla_Z V$, maps projected at the same heights for the two GB models. While the ideal GB maintains symmetric and uniform electronic features across the interface, the strained and corrugated GB exhibits a pronounced modulation of the charge distribution and the $\nabla_Z V$, particularly along the GB line, with signs of symmetry breaking and local bond rearrangements. The two boundary atoms that have been displaced downward exhibit negligible charge density at the highest tip–sample distances, due both to their vertical displacement and to the increased out-of-plane localization of their bond.

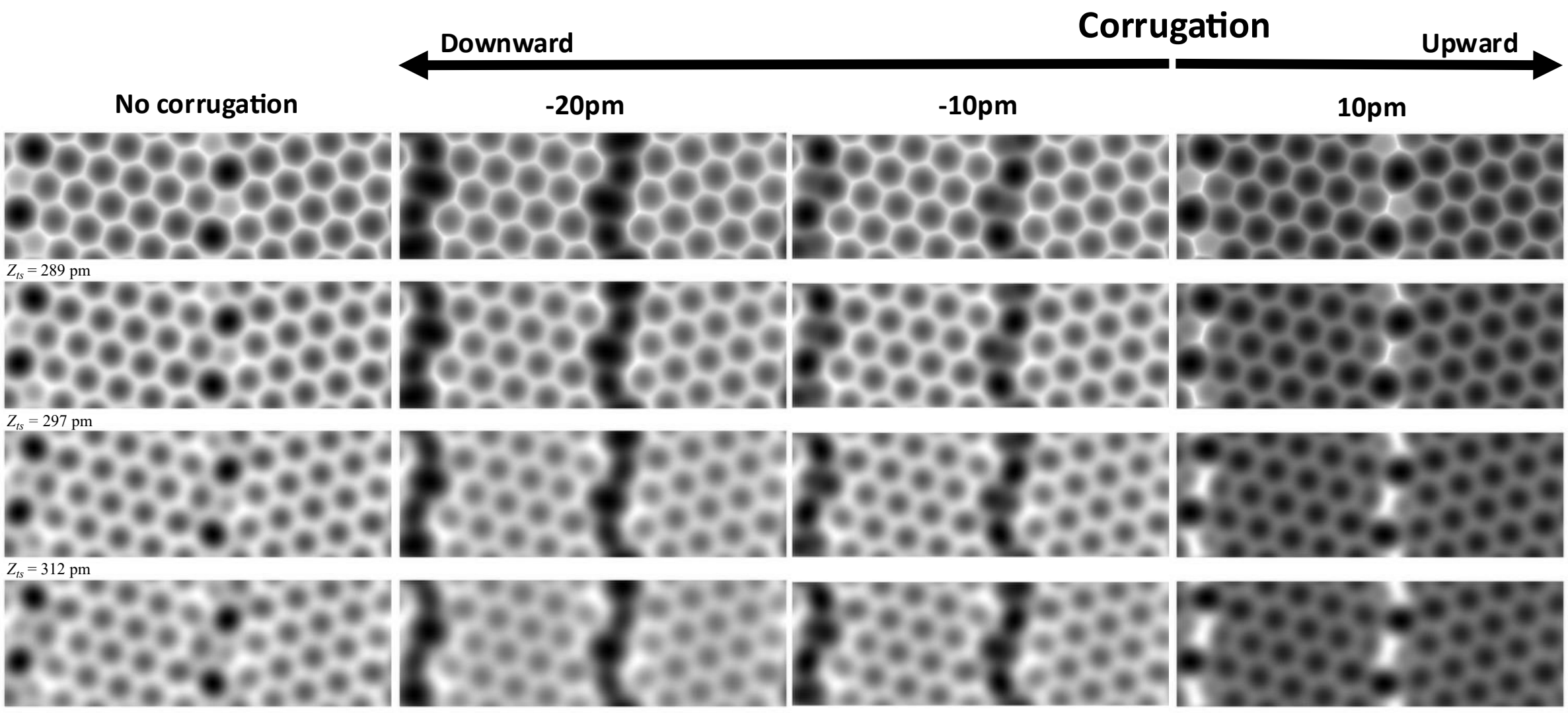

Figure S7. Simulated ncAFM contrast as a function of the corrugation. Simulated ncAFM images of a non-corrugated GB (left column) and corrugated GB for -20 pm, -10 pm and +10 pm out-of-plane displacements (minus/plus means downward/upward displacement) at different tip-sample distance (Z_{ts}). All the images correspond to an imposed compressive in-plane strain of 25 pm. The contrast in the boundary is very dependent on the corrugation. Best agreement with the experiment is found for a downward corrugation of ~ 10 pm, where features originated by the bonds of the 5-7 rings are still barely observed while the contrast on the bond between the two downward displaced atoms is missed.

Supplementary note 3: Manipulation of the metastable graphene grain boundaries

In the main text, we discussed that the observed atomic structure of the metastable graphene grain boundary can undergo modification through local manipulation (e.g., see Figure S8). In this work, such manipulation procedure consisted of repeated indentations using the ncAFM tip at different places within the GB area and around its close vicinity with the feedback loop open and maintaining the cantilever oscillation active during the whole indentation process. This indentation consisted of performing frequency shift *versus* distance ($\Delta f(z)$) curves, with typically recorded ones plotted in Figure S9 as reference. As seen, the tip-sample interaction extends well beyond the repulsive regime—likely making atomic contact—where the scanner piezo moves an average of 4-6 nm further compared to normal imaging tip-sample distances (~ 1-5 Å). This suggests that the energy exchange between the tip and sample is probably sufficiently high to facilitate surface relaxation of the GB region. As a result, the GB transitions from a strained and corrugated state to a fully flattened ideal configuration. In general, the modification does not occur gradually, but we believe in a sudden way during one of the indentations, as after repeated indentations a change in the GB area is observed, where regular pentagon and heptagon rings emerge. Once it is modified, it is impossible to reverse back to a metastable configuration, as far as we have tried during the experiments by repeating the very same manipulation procedure, although higher statistics are needed to confirm this. The existence of potential modification of the graphene grains is not observed (e.g. twist angle modification).

An example between the initial and final stage of the grain boundary region, at different fields of views, after the manipulation is shown in Figure S10, where one can clearly observe differences in the atomic structure in the GB area. Additionally, we note that these modifications are also appreciable even in the experimentally acquired raw ncAFM images, as evidenced in Figure S11.

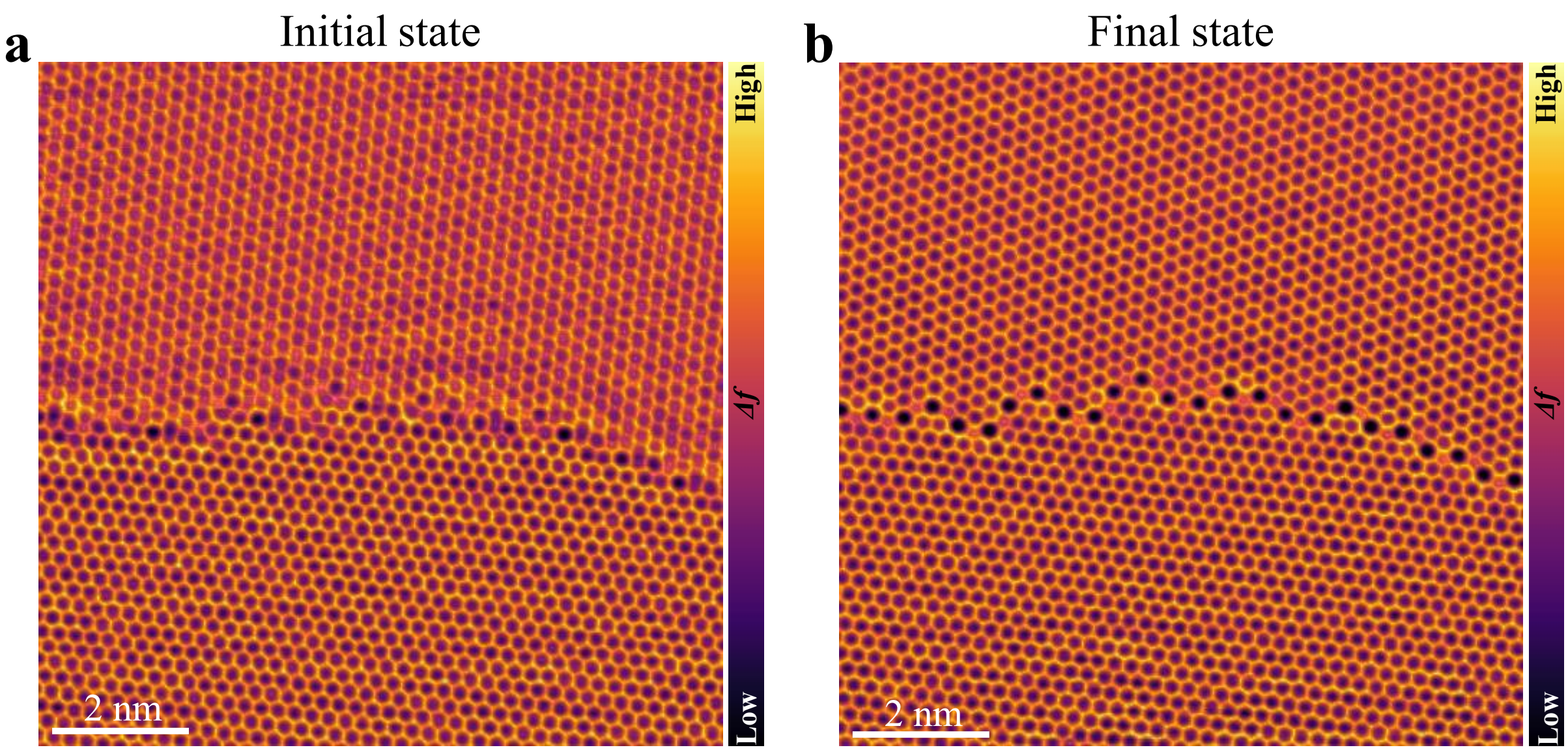

Figure S8. a,b) Corresponding enlarged view of ncAFM images shown in Figure 2a,e, revealing the GB region before and after ncAFM tip manipulation. FFT filtering and 3D effect have been applied to all the images.

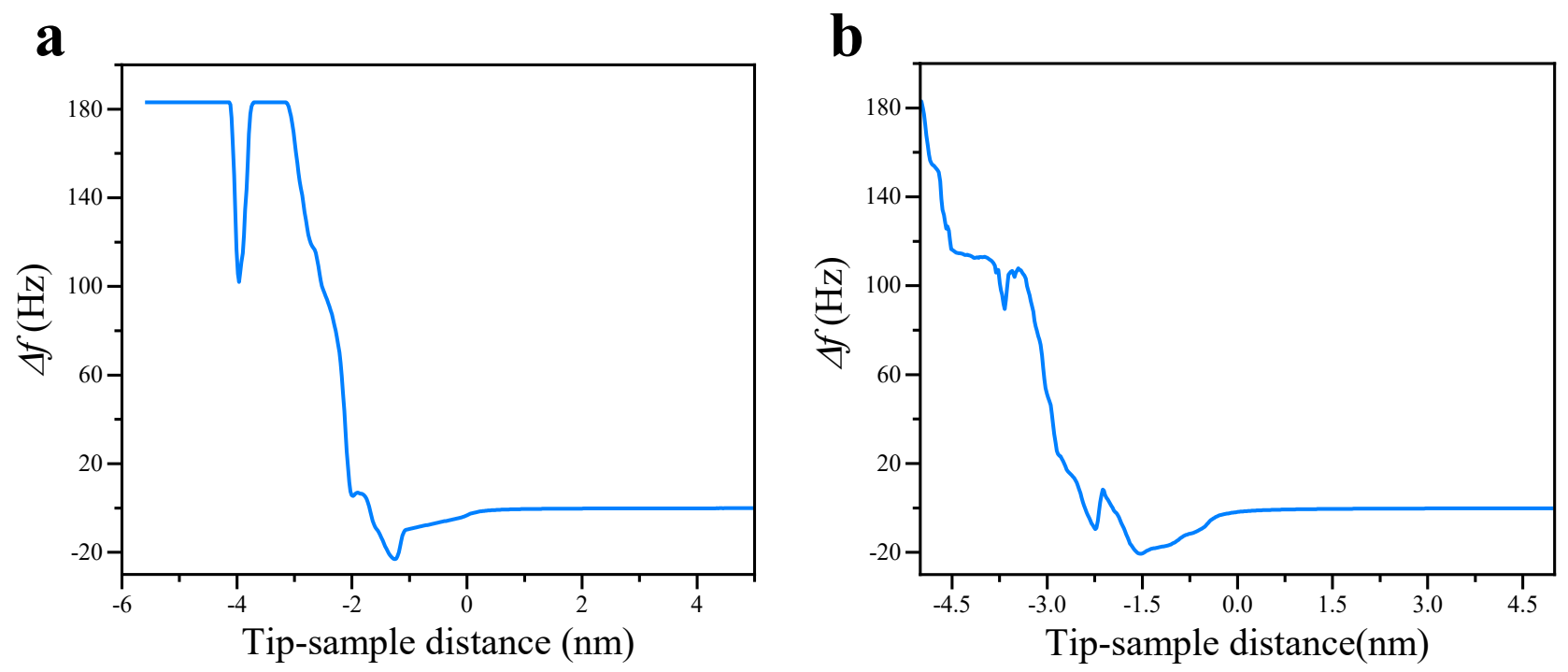

Figure S9. Typical *Δf* curves registered as function of the tip-sample distance during the application of indentation on the GB.

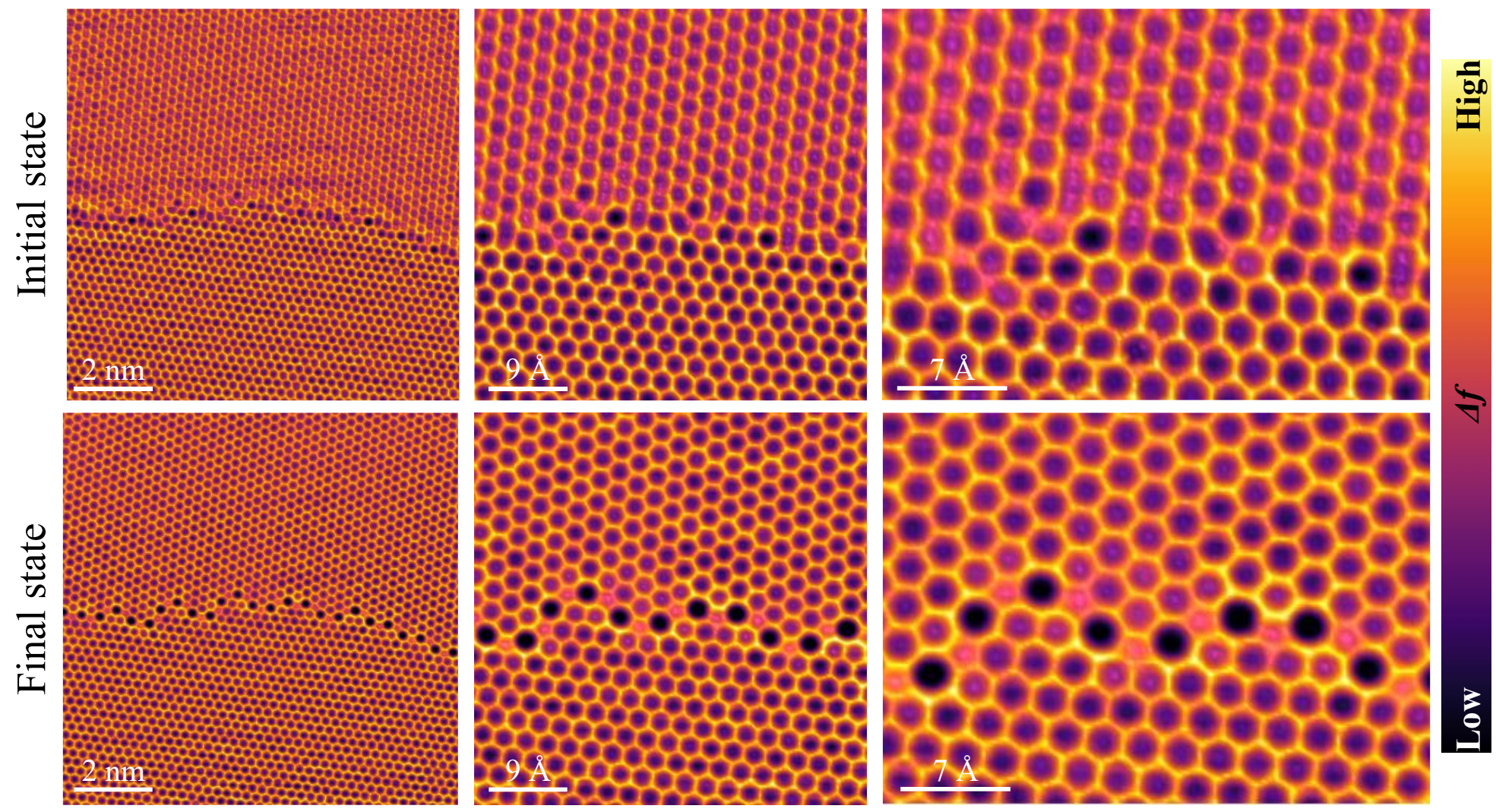

Figure S10. Different magnification constant height ncAFM images of the GB region described in Figure 2 before and after manipulation with the ncAFM tip. FFT filtering and 3D effect have been applied to all the images. FFT filtering and 3D effect have been applied to all the images.

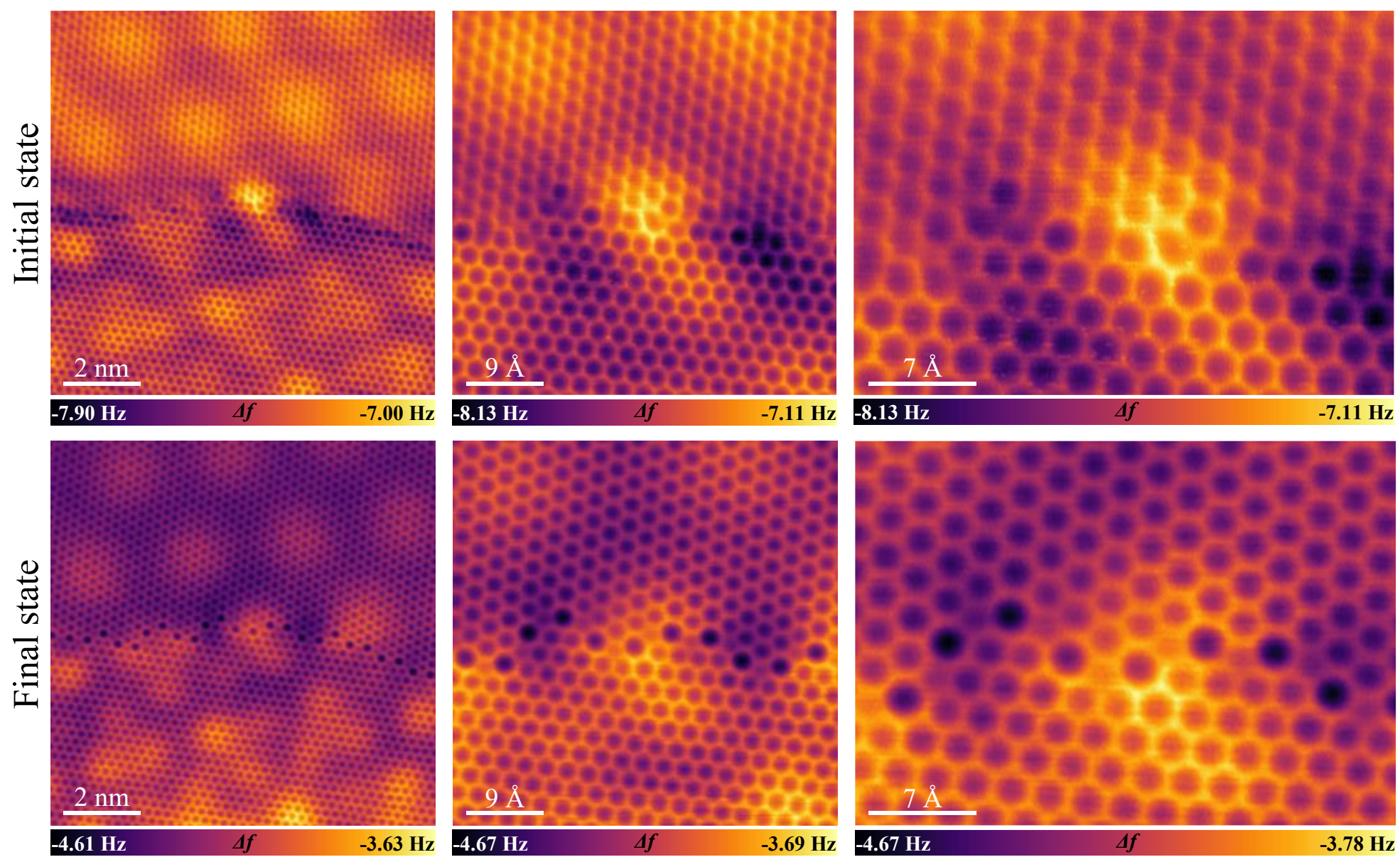

Figure S11. Experimentally measured constant height ncAFM images at different fields of view of the GB described in Figure 2 before and after manipulation with the ncAFM tip. These images are the experimental raw (without applying the filtering process) images of the one shown in Figure S10.

Supplementary note 4: Additional data regarding the coexistence of metastable and ideal GBs, and their manipulation

To further support the existence of both energetically metastable and ideal grain boundaries on the as-grown samples of graphene, in Figure S12 we show additional examples of these two types of GBs that present distinctive geometrical features along the GB area. In the same line, supporting example of the manipulation of metastable GB driven into a more stable and ideal configuration is provided in Figure S13. However, in this case, we did not determine the exact local corrugation and strain needed in the formation of the initial metastable GB.

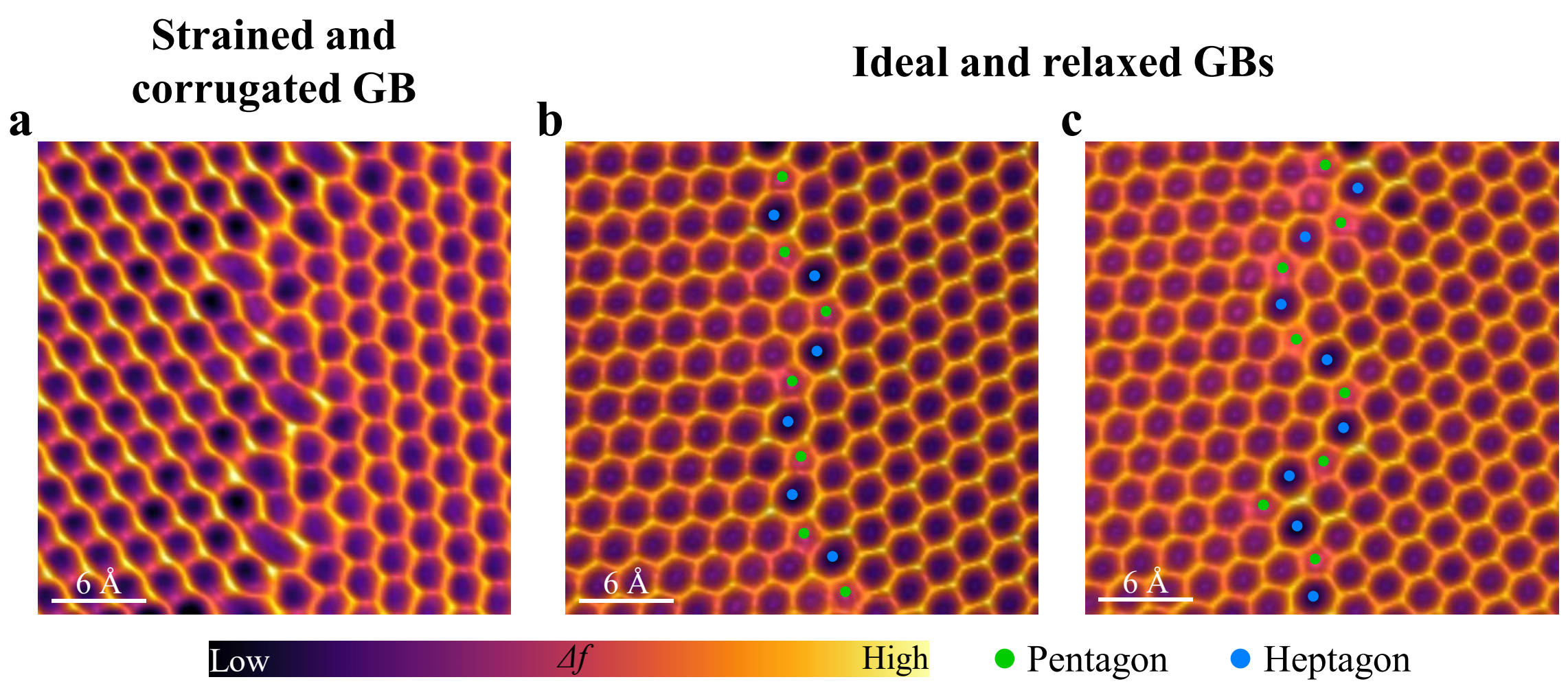

Figure S12. Additional data of strained and corrugated and ideal GBs. Representative constant height ncAFM images recorded at different sample regions showing complementary examples of strained and corrugated GB (a), as well as ideal GBs (b-c). FFT filtering and 3D effect have been applied to all the images.

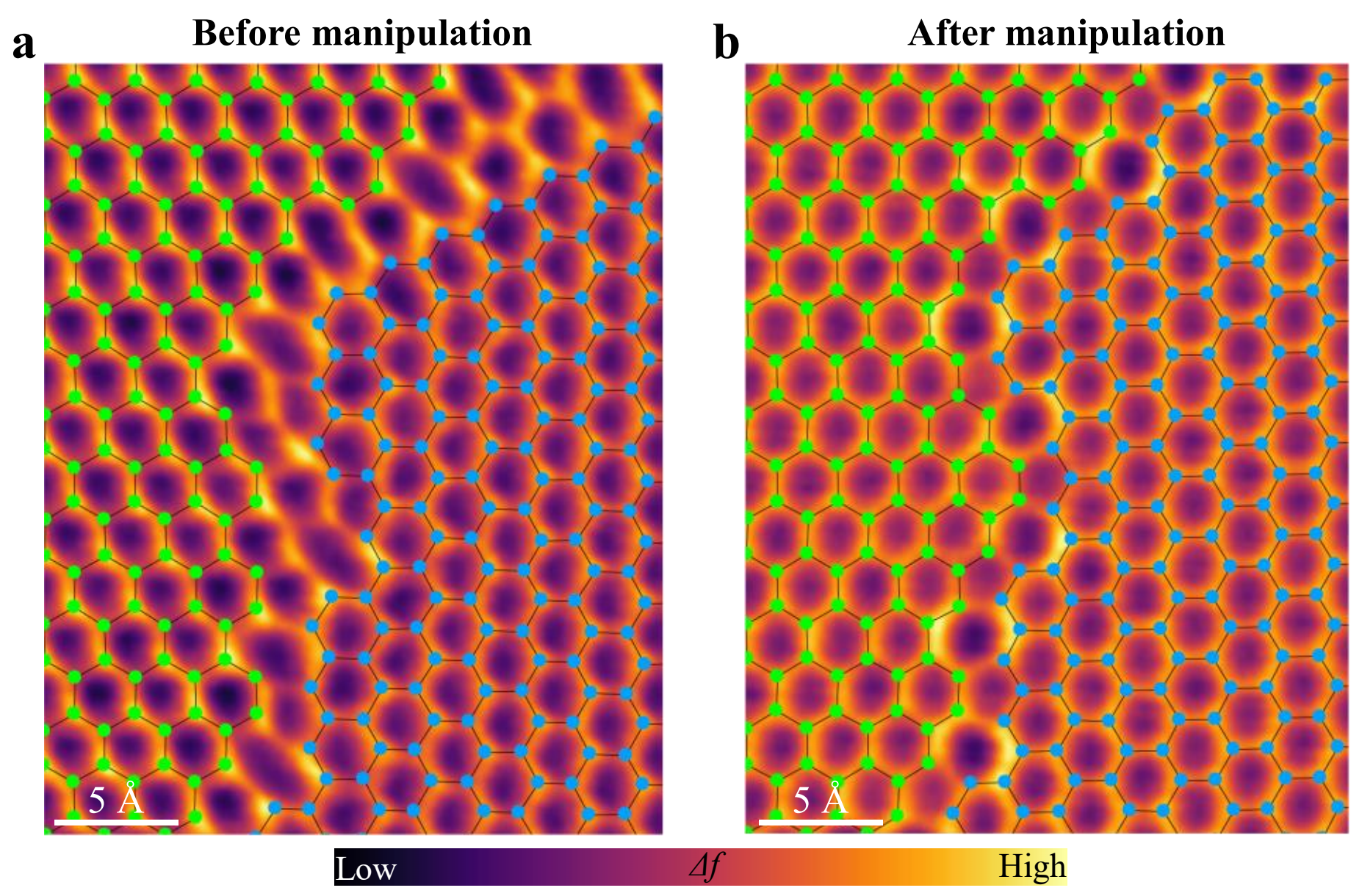

Figure S13. Further example of tip-induced manipulation of an initial metastable GB (a) driven to a stable and regular GB (b). FFT filtering has been applied to the images.

Supplementary note 5: Experimental evidence of strain in the metastable grain boundary

To experimentally sustain the presence of strain in the GB area, we superimposed the acquired ncAFM images—captured before and after ncAFM tip manipulation—onto theoretical models of the GBs, comparing strained and corrugated structures against flat and planar configurations, as illustrated in Figure S14. In both scenarios, our experimental observations exhibit a remarkable alignment with the corresponding theoretical models, reinforcing the validity of our conclusions. Additionally, we experimentally observe that the structural distortions in the metastable GBs area impact also the graphene structure over several nanometers' length scale. This is evidenced by the observed "quinoidal" configuration of the graphene honeycomb in the upper grain (see Figure S14a), where 2 out of 6 bonds are imaged which brighter contrast. This is indicative of potential differences in the C bond lengths.

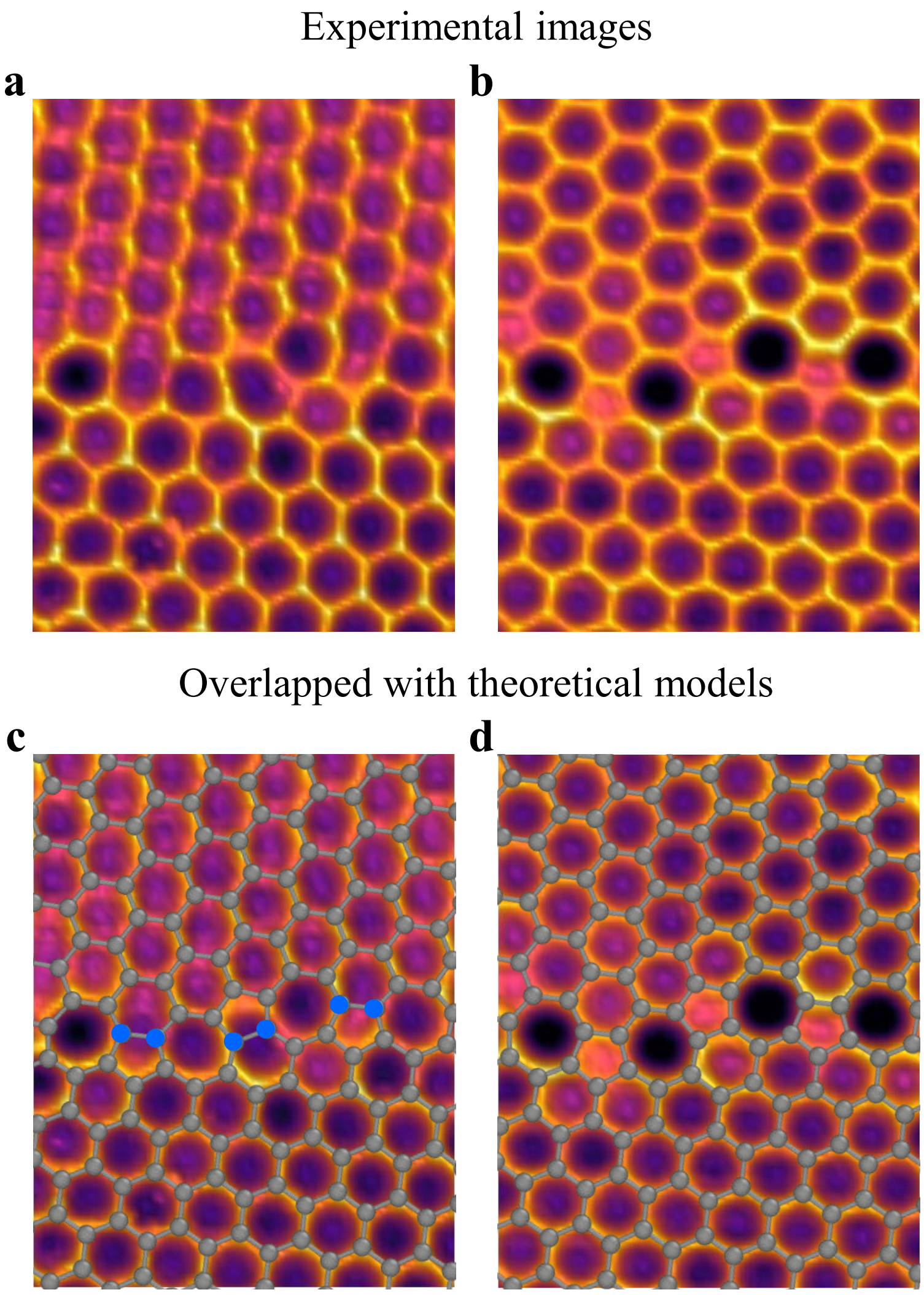

Figure S14. Comparison between the experimental acquired constant height ncAFM images (a,b) and their respective best theoretical GB model superimposed on them (c,d). FFT filtering and 3D effect have been applied to all the images. In (c), the overlapped model corresponds to a GB under the effects of compressive strain s = 25 pm and vertical corrugation h = -10 pm (lowered). Blue dots highlight the C atoms in the boundary that are under the compressive strain effect and vertical corrugation. Meanwhile, in (d), the model corresponds to an ideal GB (flat and planar configuration). These are the models used to simulate the ncAFM images depicted in Figure 2g,h.

Supplementary note 6: Morphology of the metastable grain boundary area

The induction of strain in the GB can likely be attributed to the presence of structural defects on the surface near the GB area. As shown in Figure S15, several prominent defects, such as wrinkles and beads are observed in proximity to the GB (highlighted by green squares). These defects may serve as pinning points, preventing the complete relaxation of the metastable GB structure and consequently giving rise to the asymmetric geometric arrangement of pentagonal and heptagonal rings, as discussed in Figure 2 of the main text. However, upon manipulation with the ncAFM tip, this strain is effectively erased, allowing the GB to revert to its fully relaxed planar configuration.

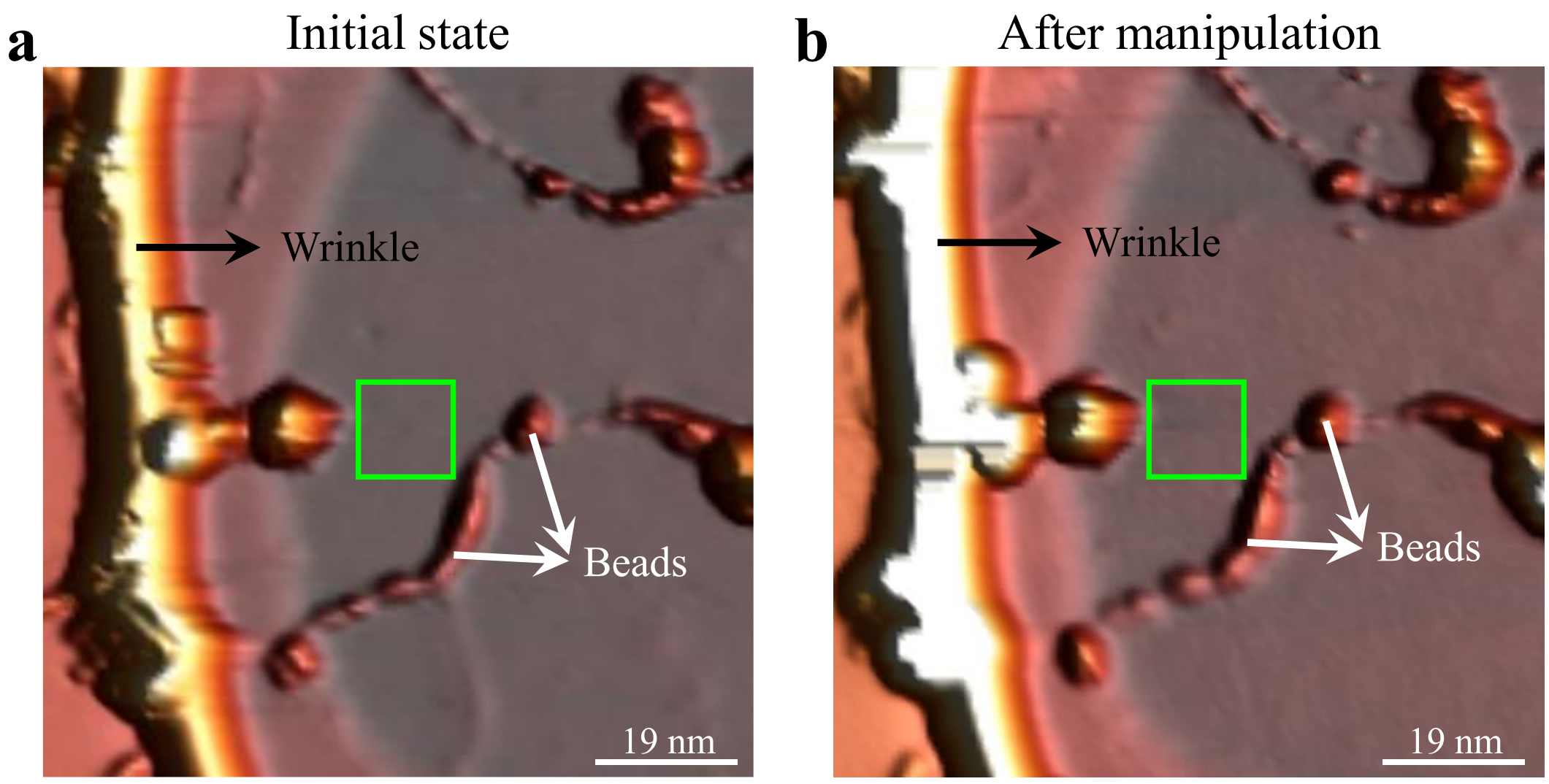

Figure S15. Large-scale topographic ncAFM images showing the morphology of the region around the GB (indicated by the green squares) before and after we performed the manipulation described in the main text. Surface defects such as wrinkles and beads are highlighted. A derivative filter has been applied to all images to enhance the features. Acquisition parameters: a) Δf = -1.5 Hz, V_{CPD} = 0 V. b) Δf = -1.5 Hz, V_{CPD} = 0 V.

Supplementary note 7: Long range perturbation and Clar's sextet theory in graphene grain boundaries

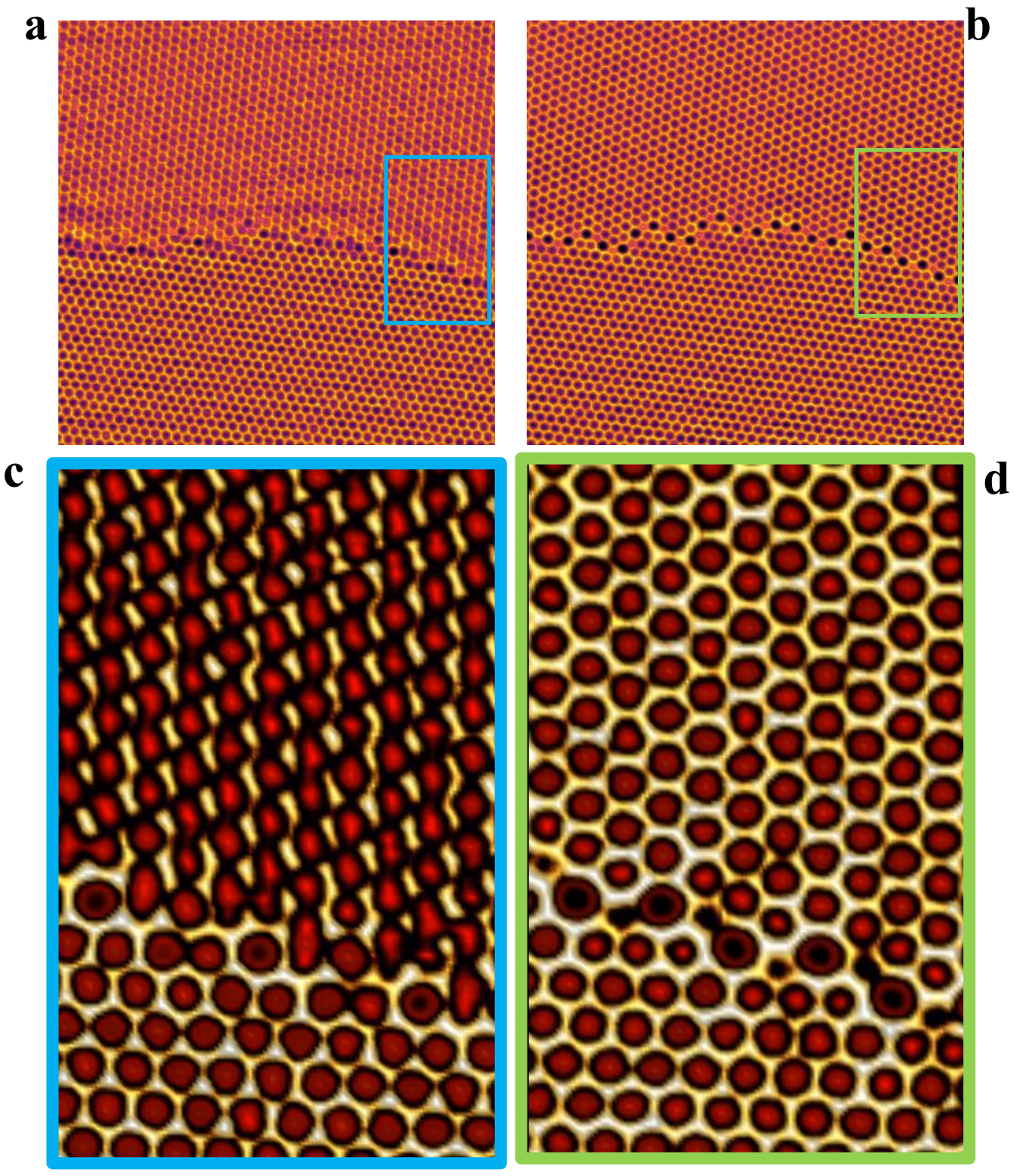

Figure S16. a,b) Same ncAFM images as Figures 2a,e of the main manuscript, acquired in the same region before and after manipulating a strained metastable GB. c,d) Zoom in of the region outlined by a blue (green) rectangle. Image contrast has been adjusted to better visualize the bond structure. In the strained GB (a,c), the upper grain displays a peculiar quinoidal bond texture in which one bond per carbon hexagon has higher intensity. On the contrary, in the relaxed grain boundary (b,d), this so-called quinoidal bond texture is not seen, and both graphene domains adopt an ideal graphene appearance

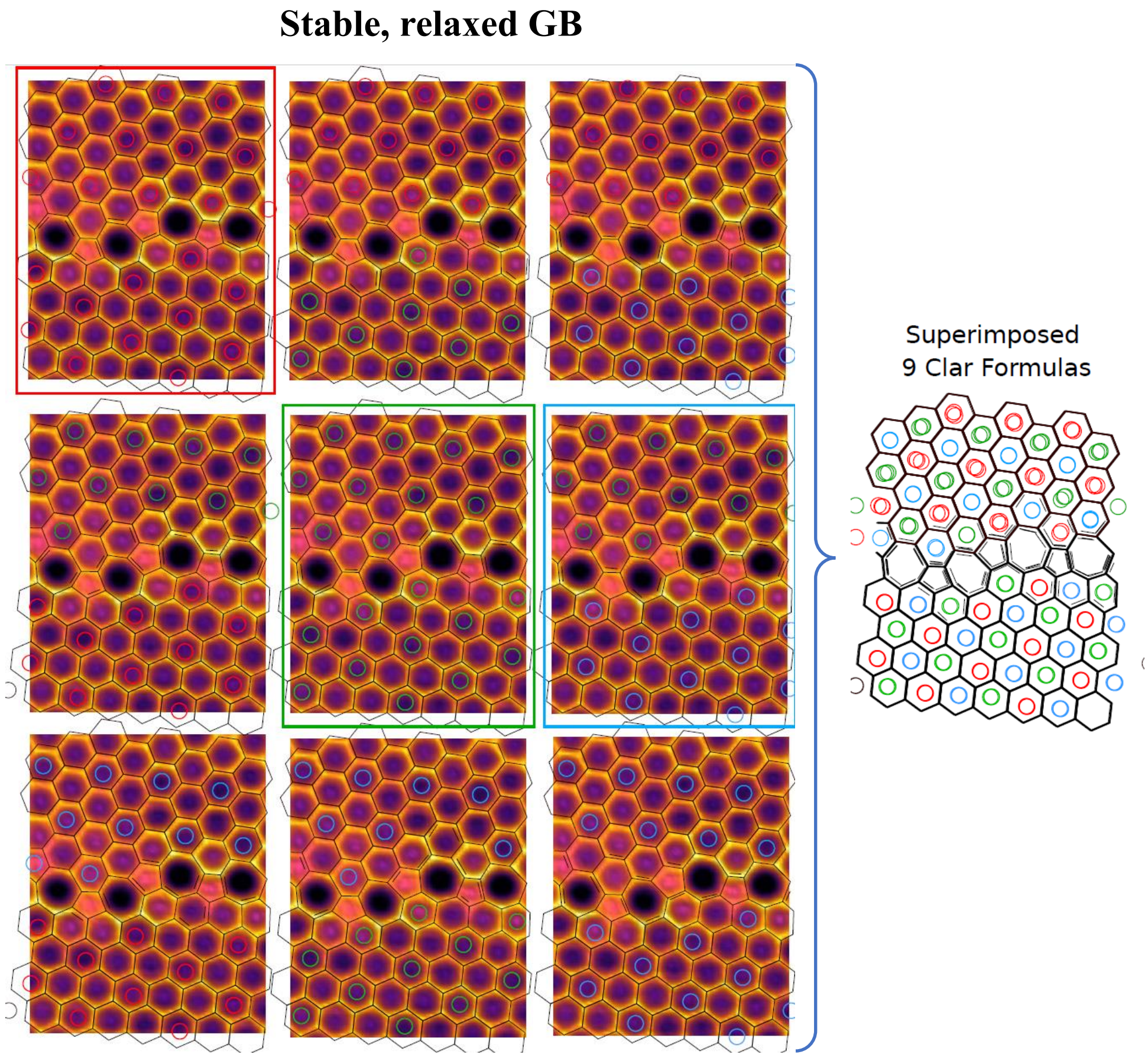

Figure S17. For the relaxed, stable grain boundary, both graphene domains adopt an all-benzenoid configuration—an ideal graphene appearance. Clar formulas that preserve the threefold degeneracy of Clar structures on either side of the boundary can be constructed, provided that the double bonds formed during grain-boundary creation remain confined within it.

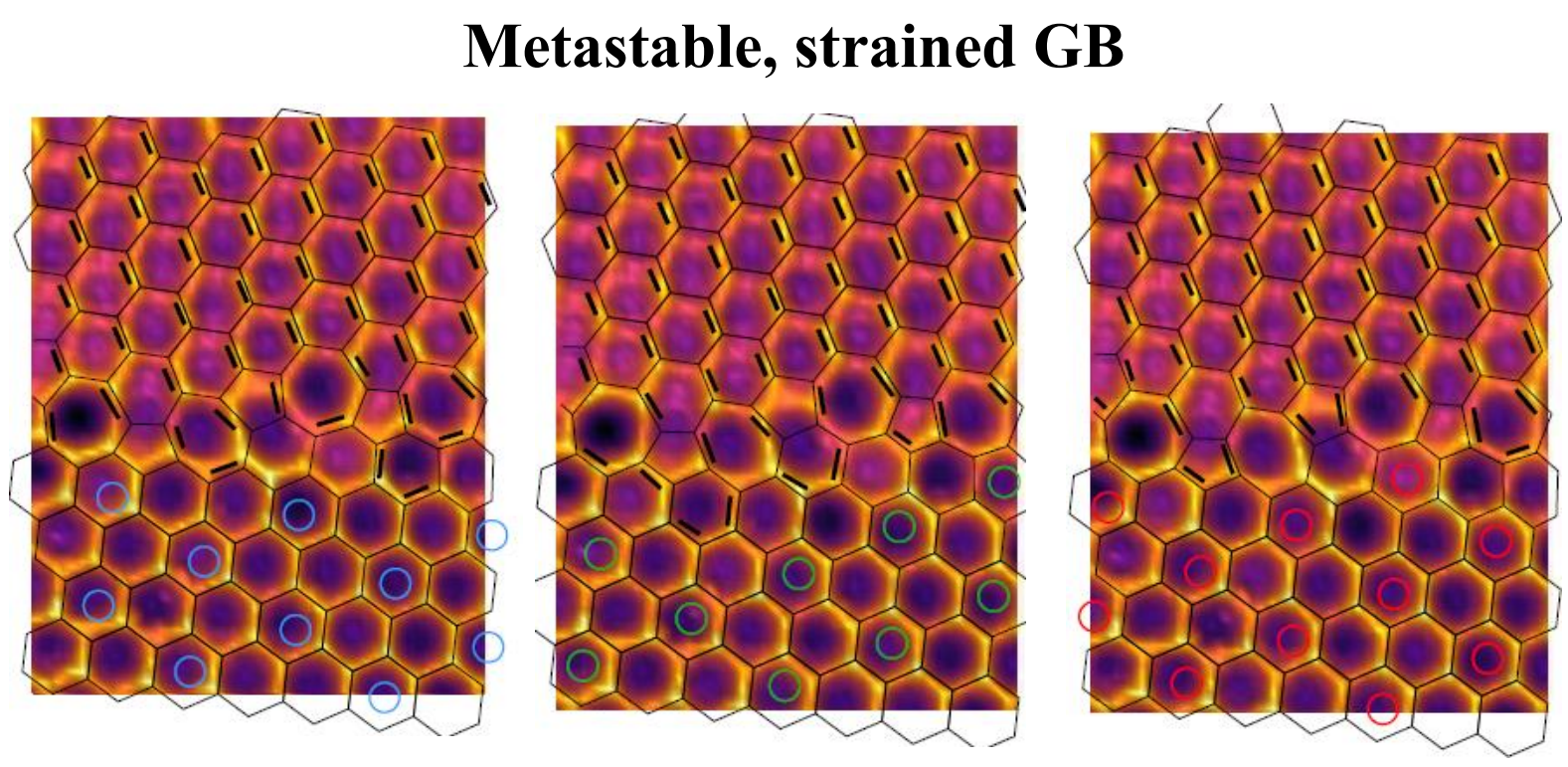

Figure S18. Clar formulas can be constructed to reproduce the quinoidal bond pattern observed in the upper grain. However, our current understanding of the grain boundary details remains insufficient to explain why this specific configuration is favored over other possible arrangements.

Supplementary note 8: Moiré patterns on Gr/SiC(000-1)

As mentioned in the Methods section, our experiments were conducted on polycrystalline graphene surfaces grown on SiC(000-1). This growth method results in the formation of multilayer graphene with significant vertical rotational disorder, which facilitates electronic decoupling between consecutive layers. Additionally, this rotational misalignment leads to the emergence of multiple moiré patterns on the surface, characterized by considerable heterogeneity in their periodic wavelengths. In Figure S19 we show several representative moiré patterns observed in the present work on the graphene/SiC(000-1) surfaces, measured using either constant *frequency shift* (Δf) or constant height ncAFM acquisition modes.

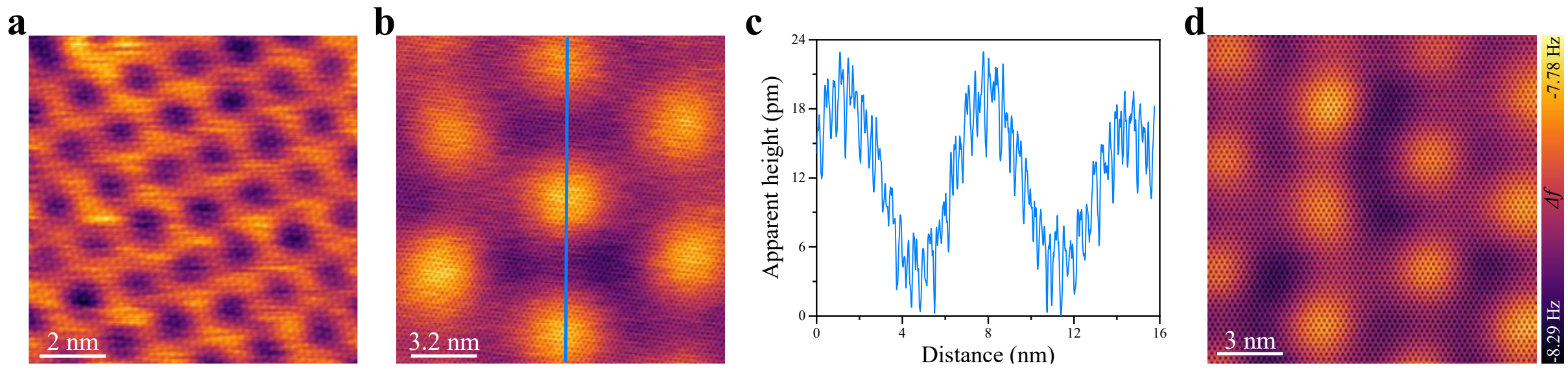

Figure S19. Graphene moiré patterns observed on the surface of Gr/SiC(000-1) by ncAFM. a-b) Atomically resolved constant *frequency shift* ncAFM images of different moiré patterns. c) Apparent height profile along the blue line drawn in (b). Despite its large periodicity (~ 7 nm), the moiré pattern shows a small apparent corrugation (~ 12 pm). d) Atomically resolved constant height ncAFM image of large periodicity moiré pattern. Acquisition parameters: a) Δf = -4 Hz, V_{CPD} = -40 mV. b) Δf = -11 Hz, V_{CPD} = 50 mV. d) V_{CPD} = 0 V.

Supplementary note 9: Conditioning of the experimental ncAFM tip-apex

In this note we detail how we carried out the conditioning of our tip-apex before performing high-resolution imaging of the atomic structure of graphene grain boundaries with our ncAFM. In Figure S20 we show two representative ncAFM topographic images revealing the overall surface morphology of the Gr/SiC(000-1) samples studied in this work. We observe the existence of several surface imperfections, such as structural defects like wrinkles and beads, and aggregates of contaminants, whose chemical composition is unknown. Indeed, we prepare our tip-apex on top of these contaminants by performing indentation (*Δf(z)* spectroscopy) or continuous scanning on top of these aggregates until a sudden enhancement of the spatial resolution is observed in topography images. Then, we move the tip to the preselected GB areas (like those highlighted in Figure S20) to carry out the high-resolution imaging of their atomic structure. Although we don't know the exact chemical nature of our tip-apex, we note that this procedure has allowed us to measure all the bond-resolved ncAFM images shown in this work routinely. Therefore, this procedure is empirically reliable and our simulations using a proxy CO tip reproduce the experimental contrast. However, we highlight that there could be some experimental constraints in the future when these unknown contaminants are not present.

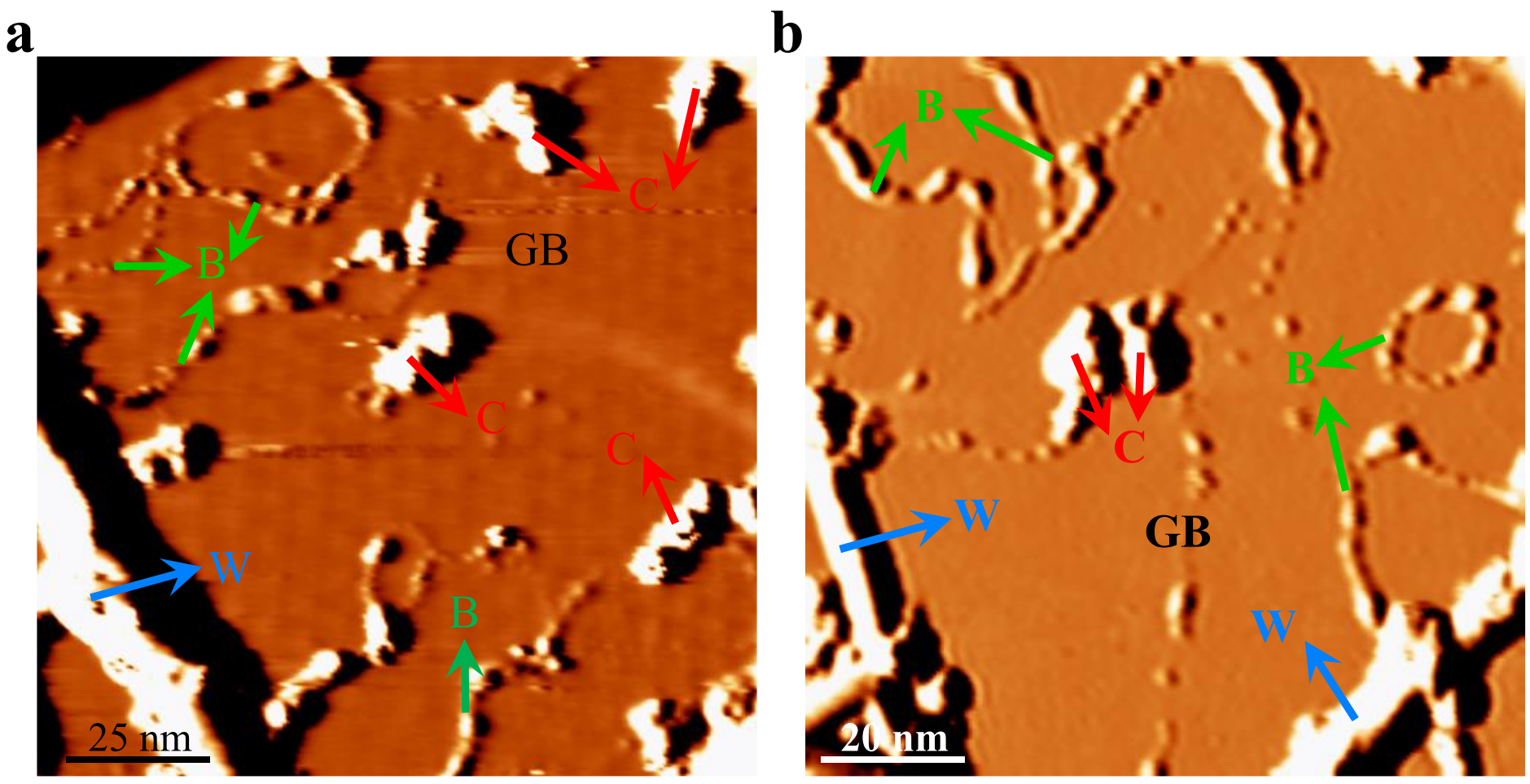

B ≡ Beads W ≡ Wrinkles C ≡ Contaminants GB ≡ Grain boundary

Figure S20. General surface morphology of our Gr/SiC(000-1) samples studied in the present work. The main surface imperfections are labelled accordingly. A derivative filter has been applied to all images to enhance the visibility of the features. Acquisition parameters: a) Δf = -4 Hz, V_{CPD} = 130 mV. b) Δf = -0.8 Hz, V_{CPD} = -40 mV.

Supplementary note 10: Graphene grain boundaries visualized in topographic ncAFM and STM imaging mode

In principle, graphene grain boundaries can be characterized using various scanning probe microscopy techniques and their different imaging modes. In this study, we demonstrate that the intrinsic chemical structure of these boundaries has been successfully imaged only through the constant height imaging mode of ncAFM, following tip-apex conditioning as described in Supplementary note 9. Notably, this structure remains inaccessible via constant *frequency shift* (topographic) ncAFM or constant current (topographic) STM imaging modes. This limitation is evident in Figure S21 and Figure S22, where we show the grain boundary as imaged in both the topographic imaging mode of ncAFM and STM.

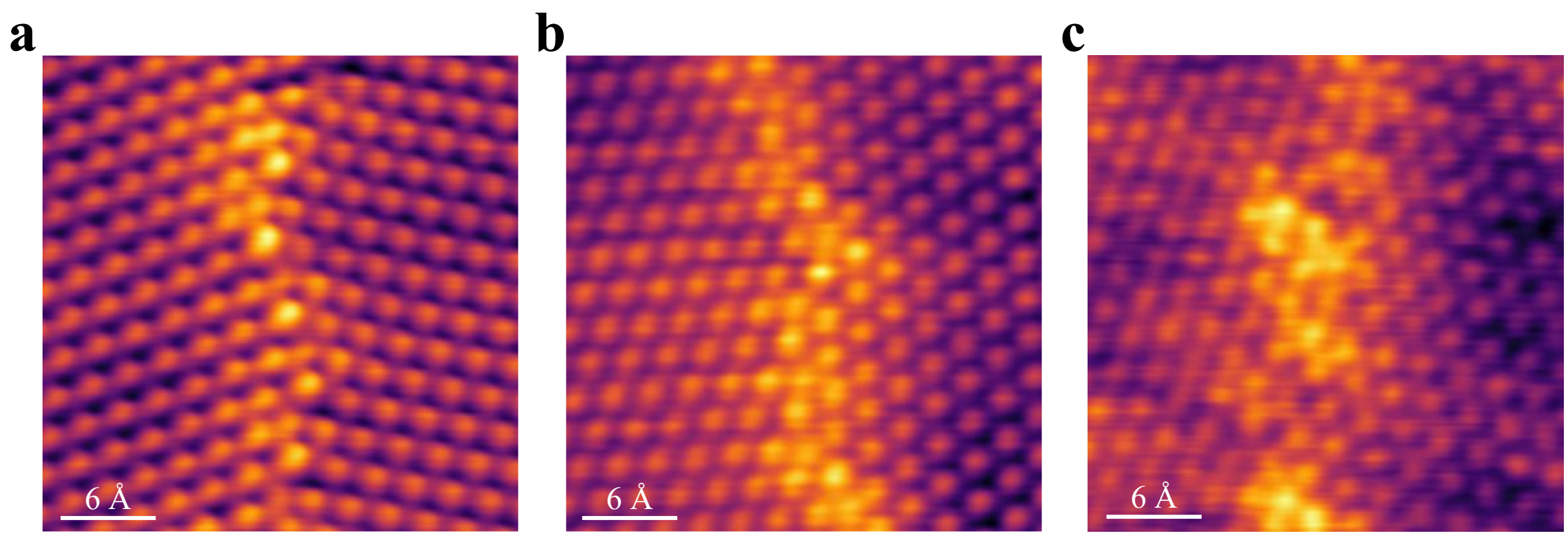

Figure S21. Graphene grain boundaries visualized in constant Δf imaging mode. a-c) Representative topographic ncAFM images acquired at different GB regions of the sample. As can be observed, the chemical structure of the grain boundaries can not be resolved in this measurement mode. The misorientation angle between the graphene domains is: (a) 31º, (b) 30º, and (c) 35º.

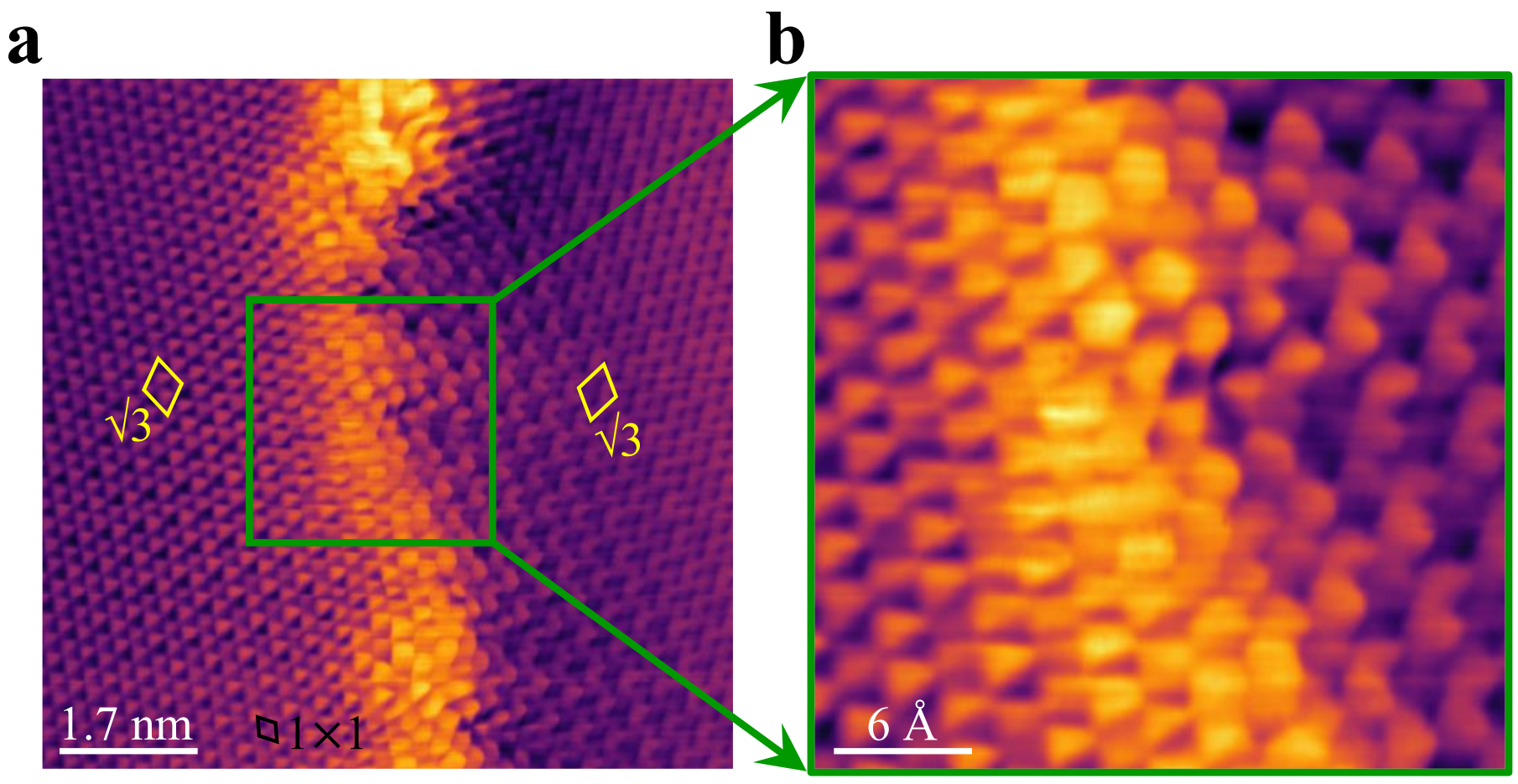

Figure S22. Graphene grain boundary observed by STM. a) Constant current STM image of a grain boundary showing intervalley scattering of graphene quasiparticles, evidenced by a (√3×√3)-R30º interference pattern relative to the graphene (1×1) lattice. b) Magnified STM image within the green square region in (a). The chemical structure of GB is unresolved due to STM probes the LDOS, which is dominated by the grain boundary's electronic structure. Acquisition parameters: a,b) V_s = -200 mV, I_t = 0.18 nA. c) V_{CPD} = -40 mV.
